# Scaffold coupling: ERK activation by trans-phosphorylation across different scaffold protein species

**DOI:** 10.1126/sciadv.add7969

**Published:** 2023-02-15

**Authors:** Ana Martín-Vega, Laura Ruiz-Peinado, Rocío García-Gómez, Ana Herrero, Dalia de la Fuente-Vivas, Swetha Parvathaneni, Rubén Caloto, Marta Morante, Alex von Kriegsheim, Xosé R. Bustelo, David B. Sacks, Berta Casar, Piero Crespo

**Affiliations:** ^1^ Instituto de Biomedicina y Biotecnología de Cantabria (IBBTEC), Consejo Superior de Investigaciones Científicas (CSIC) - Universidad de Cantabria, Santander 39011, Spain.; ^2^ Centro de Investigación Biomédica en Red de Cáncer (CIBERONC), Instituto de Salud Carlos III, Madrid 28029, Spain.; ^3^ Department of Laboratory Medicine, National Institutes of Health, Bethesda, MD 20892, USA.; ^4^ Centro de Investigación del Cáncer and Instituto de Biología Molecular y Celular del Cáncer, CSIC-Universidad de Salamanca, Salamanca 37007, Spain.; ^5^ Edinburgh Cancer Research UK Centre, MRC Institute of Genetics and Molecular Medicine, University of Edinburgh, Edinburgh EH4 2XU, UK.; ^6^ Department of Medicine, Georgetown University, 3700 O St NW, Washington, DC 20057, USA.; ^7^ Department of Pathology, George Washington University, 2121 I St NW, Washington, DC 20052, USA.; ^8^ University of Cape Town, UCT Faculty of Health Sciences, Barnard Fuller Building, Anzio Rd, Observatory, Cape Town, 7935 South Africa.

## Abstract

RAS-ERK (extracellular signal–regulated kinase) pathway signals are modulated by scaffold proteins that assemble the components of different kinase tiers into a sequential phosphorylation cascade. In the prevailing model scaffold proteins function as isolated entities, where the flux of phosphorylation events progresses downstream linearly, to achieve ERK phosphorylation. We show that different types of scaffold proteins, specifically KSR1 (kinase suppressor of Ras 1) and IQGAP1 (IQ motif-containing guanosine triphosphatase activating protein 1), can bind to each other, forming a complex whereby phosphorylation reactions occur across both species. MEK (mitogen-activated protein kinase kinase) bound to IQGAP1 can phosphorylate ERK docked at KSR1, a process that we have named “trans-phosphorylation.” We also reveal that ERK trans-phosphorylation participates in KSR1-regulated adipogenesis, and it also underlies the modest cytotoxicity exhibited by KSR-directed inhibitors. Overall, we identify interactions between scaffold proteins and trans-phosphorylation as an additional level of regulation in the ERK cascade, with broad implications in signaling and the design of scaffold protein–aimed therapeutics.

## Teaser

Phosphorylation reactions through the ERK cascade as regulated by scaffold proteins can occur across distinct types of scaffolds.

## INTRODUCTION

Extracellular signal-regulated kinase 1/2 (ERK, hereafter) mitogen-activated protein kinases are principal actors in the transduction of signals conveyed by external stimuli to the interior of the cell, where they switch-on biochemical processes and genetic programs fundamental to the regulation of proliferation, differentiation, and survival, among other key cellular processes (*1*, *2*). ERK signals are modulated by different types of regulatory proteins. Among these, scaffold proteins represent the most abundant, diverse, and widespread class. Scaffold proteins link the components of the sequential tiers that comprise the ERK signaling cascade to form a multienzymatic complex, by which ERK signals are fine-tuned with respect to amplitude, intensity, and duration, and afford signal fidelity by shielding the complex from interferences. In addition, scaffolds provide spatial selectivity to ERK signals by regulating their activity in a sublocalization-specific fashion (*3*–*7*).

Currently, more than a dozen protein species are considered bona fide ERK scaffold proteins. Among these is KSR1 (kinase suppressor of RAS 1), the first mammalian scaffold described as a protein binding to V-RAF murine sarcoma viral oncogene homologues C and B (C/BRAF), mitogen-activated protein kinase kinase (MEK) 1/2, and ERK1/2 (*8*) that, together with KSR2, constitutes the best-studied scaffold family. KSR1 is a ubiquitous, multidomain, cytoplasmic protein that rapidly translocates to the plasma membrane upon activation to regulate RAS-ERK signals therein, particularly at cholesterol-rich microdomains (*9*, *10*). KSR1 depletion markedly impairs RAS-ERK signaling, thereby forestalling RAS-driven oncogenic processes (*11*). This has prompted the development of small molecules aimed at KSR to be used for antineoplastic therapy (*12*, *13*).

Another well-studied scaffold is IQGAP1 (IQ motif-containing guanosine triphosphatase activating protein 1), which, together with the isoforms IQGAP 2 and 3, make up a defined family of high–molecular weight, multidomain proteins involved in a broad spectrum of signaling routes and cellular processes, particularly in the control of cell migration via cytoskeletal regulation (*14*). IQGAP1 binds to BRAF, MEK1/2, and ERK1/2, regulating signal flux, particularly in response to tyrosine kinase receptors such as epidermal growth factor receptor (EGFR) and insulin-like growth factor receptor (IGFR) (*15*, *16*). As in the case of KSR1, IQGAP1 depletion or blockade of its scaffold functions prevents RAS-ERK pathway–driven neoplasia (*17*).

Scaffold proteins are believed to function by optimizing the phosphorylation reactions occurring along the different tiers of the ERK cascade. Initially, it was proposed that scaffolds are constitutively bound to MEK1/2, and upon stimulation, unphosphorylated ERK1/2 would be incorporated into the complex to be phosphorylated therein by resident MEK (which we shall refer to as cis-phosphorylation), and then released (*3*). Subsequently, we demonstrated that ERK can also bind constitutively to scaffolds, to be cis-phosphorylated therein by scaffold-bound MEK that would enable its binding to a free, phosphorylated ERK monomer to form a dimer. Thus, scaffold proteins would act as ERK dimerization platforms (*10*, *18*–*20*). However, although our knowledge of scaffold proteins has grown substantially over the past years, the precise mechanisms whereby scaffolds facilitate ERK phosphorylation are not fully understood. For instance, it has been shown that KSR mutants deficient for binding MEK can still incorporate phosphorylated ERK in response to stimulation (*21*, *22*).

Upon investigating such conundrum, we have found that different scaffold species, namely, KSR1 and IQGAP1, can functionally associate with each other. As a consequence, ERK docked at KSR1 can be phosphorylated by MEK bound to IQGAP1, a process that we have named “trans-phosphorylation”. In this respect, we unveil that ERK trans-phosphorylation can explain the modest cytotoxic activity exhibited by KSR-directed small-molecule inhibitors. Overall, the results presented here identify interactions between scaffold proteins as a higher-order level of regulation in the ERK cascade, with broad implications in signal transduction.

## RESULTS

### In KSR1 C809Y, ERK is phosphorylated by a trans-acting MEK

**Fig. 1.**
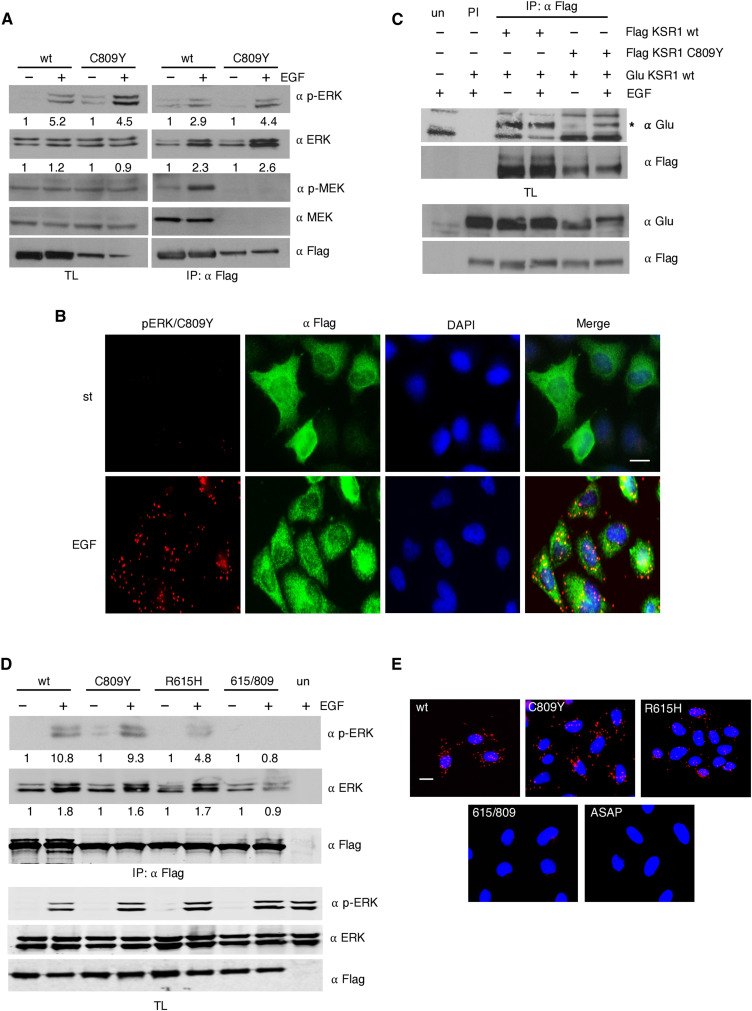
Trans-acting MEK phosphorylates KSR1 C809Y. (**A**) KSR1 C809Y incorporates phosphorylated ERK. HEK293T cells were transfected with Flag-tagged wild-type (wt) or C809Y KSR1 (1.5 μg) and stimulated with EGF (50 ng/ml, 5 min) where indicated (+) after 18 hours of starvation (−). KSR1-associated proteins were determined by coimmunoprecipitation upon anti-Flag immunoprecipitation (IP: Flag) and subsequent Western blotting. TL, total lysate. (**B**) C809Y binds to phosphorylated ERK in live cells. Ectopic Flag-tagged C809Y interaction with endogenous ERK, determined by PLA in HeLa cells after starvation, in starved (st) or EGF-treated cells. Scale bar, 10 μm. (**C**) C809Y can homodimerize. HEK293T cells were transfected with the indicated Glu- and Flag-tagged KSR1 constructs and EGF-stimulated where indicated (+). Immunoprecipitations performed with a specific antibody (IP) or with preimmune serum (PI). un, untransfected cells. (**D**) KSR1 double mutant fails to bind phosphorylated ERK. Coimmunoprecipitation assay in HEK293T cells transfected with the indicated Flag-tagged KSR1 constructs, in starved cells (−) or upon EGF stimulation where indicated (+). (**E**) KSR1 double mutant fails to bind phosphorylated ERK in vivo. HeLa cells were transfected with the indicated KSR1 constructs (2 μg). PLA as in (B), in EGF-stimulated cells. Scale bar, 10 μm. (A and D) Figures show signal intensity relative to the levels found in untreated cells. All the results shown are representative of three to five independent experiments.

Previous studies have demonstrated that the KSR1 MEK binding-deficient mutant C809Y can incorporate phosphorylated ERK in the presence of activated RAS and can efficiently mediate in RAS-induced senescence in mouse embryonic fibroblasts (MEFs) (*21*). We found that this was also the case in human embryonic kidney (HEK) 293T cells in response to EGF stimulation, where C809Y associated to phosphorylated ERK and incremented its total ERK content as a consequence of ERK dimerization (*18*), as efficiently as wild-type KSR1 (Fig. 1A). These results obtained by coimmunoprecipitation analyses were further demonstrated in vivo, by means of proximity ligation assays (PLAs) performed in HeLa cells. By this method, it was verified that ectopically expressed C809Y could associate with endogenous ERK and that this interaction mainly took place in the cytoplasm (Fig. 1B and fig. S1B).

As an explanation for this phenomenon, we contemplated two hypotheses: C809Y-associated ERK could be phosphorylated by free, cytoplasmic MEK. Alternatively, because KSR has been shown to dimerize (*23*), it would be possible that C809Y-bound ERK was phosphorylated in trans by MEK docked at a homodimerizing wild-type KSR1 or a heterodimerizing KSR2. We observed that C809Y retained its full capacity for homodimerization with wild-type KSR1, particularly under EGF stimulation (Fig. 1C). To distinguish between these alternatives, we used KSR1 R615H, a mutant form impaired for dimerization (*23*, *24*), and a double mutant, R615H/C809Y, deficient in both dimerization and MEK binding. When transfected into 293T cells, R615H retained the ability to bind phosphorylated ERK in response to EGF stimulation, although to a lower extent than C809Y, probably because this mutation also hinders KSR interaction with RAF (*23*). Conversely, no phosphorylated ERK was found associated with the 615/809 double mutant. Similarly, total ERK levels bound to the double mutant were unaltered (Fig. 1D). These results were also validated in vivo by PLA in HeLa cells. This assay demonstrated that the KSR1 dimerization–defective construct, but not the double mutant, could bind to endogenous, phosphorylated ERK in the cytoplasm. As an additional control, we used a mutant incapable of binding ERK (KSR1 ASAP), which, as expected, also failed to bind phosphorylated ERK (Fig. 1E and fig. S1E). These results suggested that C809Y-bound phosphorylated ERK would result from the action of a trans-acting MEK associated to a dimerizing wild-type KSR molecule, not from free, soluble MEK, as this form should be capable of phosphorylating ERK bound to the KSR1 double mutant. Consistently, we have termed this process trans-phosphorylation.

### KSR1 binds to IQGAP1

**Fig. 2.**
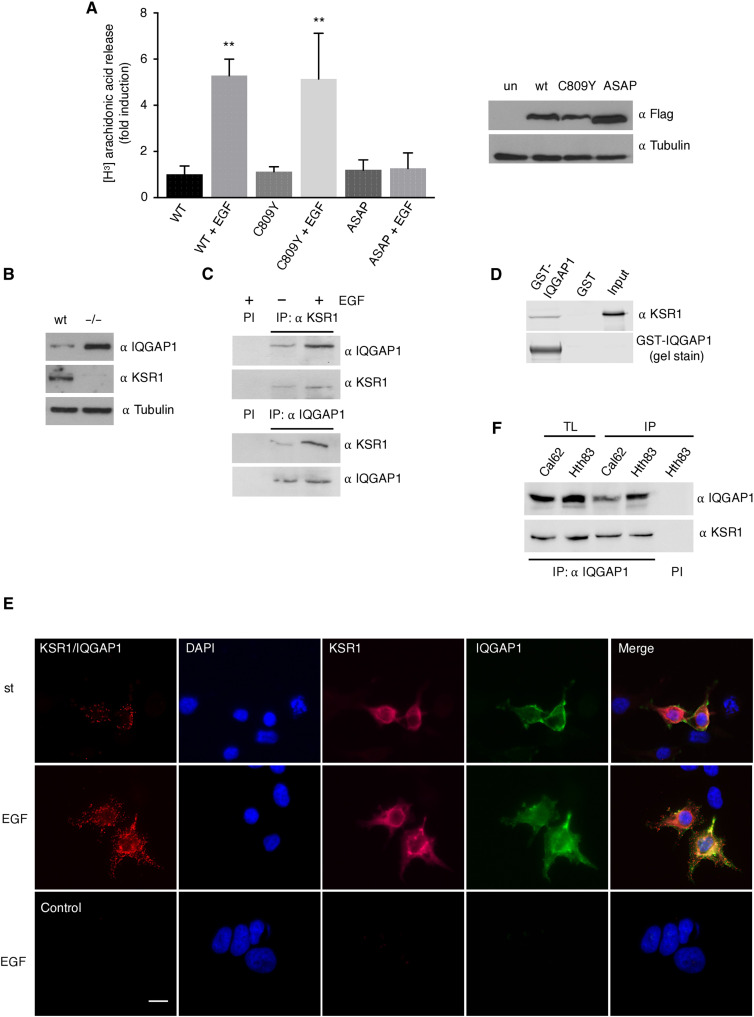
KSR1 binds IQGAP1. (**A**) KSR1 C809Y is active in KSR1^−/−^ MEFs. Left: cPLA_2_ activity in KSR1^−/−^ MEFs transfected with the indicated KSR1 constructs (2 μg) under starvation conditions and after EGF stimulation (50 ng/ml, 5 min). Data show means ± SEM for two independent experiments, relative to the value of KSR1 wt. *P* values: **P < 0.01 by two-tailed unpaired Student’s *t* test. Right: KSR1 construct expression levels. (**B**) IQGAP1 expression in wt and KSR^−/−^ MEFs. (**C**) IQGAP1 and KSR1 interaction in vivo. Endogenous IQGAP1 or KSR1 was immunoprecipitated from HEK293T, starved for 18 hours (−), or EGF-stimulated, and the coimmunoprecipitating scaffold was detected by WB. PI, immunoprecipitation using preimmune serum. (**D**) IQGAP1 and KSR1 interaction in vitro. Pull-down using glutathione *S*-transferase (GST)–IQGAP1 with a bacterially expressed, purified, full-length KSR1. (**E**) IQGAP1 and KSR1 interaction in live cells. Association of ectopic Flag-KSR1 and Myc-IQGAP1 (1 μg each), determined by PLA in HeLa cells after starvation, in starved (st) or EGF-treated cells. Control, untransfected cells. Scale bar, 10 μm (see also fig. S1). (**F**) Association of endogenous KSR1 and IQGAP1 in the indicated proliferating tumor cell lines, as determined by coimmunoprecipitation upon anti-IQGAP1 immunoprecipitation (IP). All the results shown are representative of three to five independent experiments.

We reasoned that if C809Y incorporated phosphorylated ERK via trans-phosphorylation from MEK bound to a dimerizing KSR 1 or 2, in a cellular context devoid of these scaffolds, C809Y would not harbor phosphorylated ERK. We have previously demonstrated that cytosolic phospholipase A_2_ (PLA_2_) is activated by ERK when its signals are specifically scaffolded by KSR1. As such, in KSR1^−/−^ MEFs, which do not express KSR2 either (*25*), EGF did not induce PLA_2_ activation, but it could be rescued by KSR1 ectopic expression (*10*). Following this logic, in the same setting, the KSR1 C809Y mutant should be incapable of such a rescue. To our surprise, C809Y facilitated EGF-induced PLA_2_ activation as efficiently as wild-type KSR1, whereas the ASAP mutant, defective for binding ERK, did not (Fig. 2A).

In light of these data, the possibility existed that some other scaffold protein species could also intervene on KSR1 C809Y–bound ERK trans-phosphorylation. With the aim of identifying such scaffolds, mass spectrometry analyses were carried out to unbiasedly detect proteins that associated with KSR1. These analyses identified IQGAP1 as a KSR1-interacting protein (fig. S2A). IQGAP1 expression levels were found to be markedly up-regulated in KSR1^−/−^ MEFs compared to the wild-type cells (Fig. 2B). To validate the KSR1-IQGAP1 interaction, we performed coimmunoprecipitation assays in HEK293T cells, which demonstrated that endogenous IQGAP1 and KSR1 readily associated with each other, more so upon EGF stimulation (Fig. 2C). In vitro analysis using purified proteins expressed in bacteria showed that the binding between KSR1 and IQGAP1 was direct (Fig. 2D). Moreover, PLA assays revealed an association between KSR1 and IQGAP1 in living cells, which was enhanced EGF stimulation, particularly at the cell periphery (Fig. 2E and fig. S2B). Endogenous IQGAP1 and KSR1 also associated with each other in the tumor cell lines of thyroid origin Cal62 and Hth83 (Fig. 2F), demonstrating that this interaction is widespread among different types of cells.

**Fig. 3.**
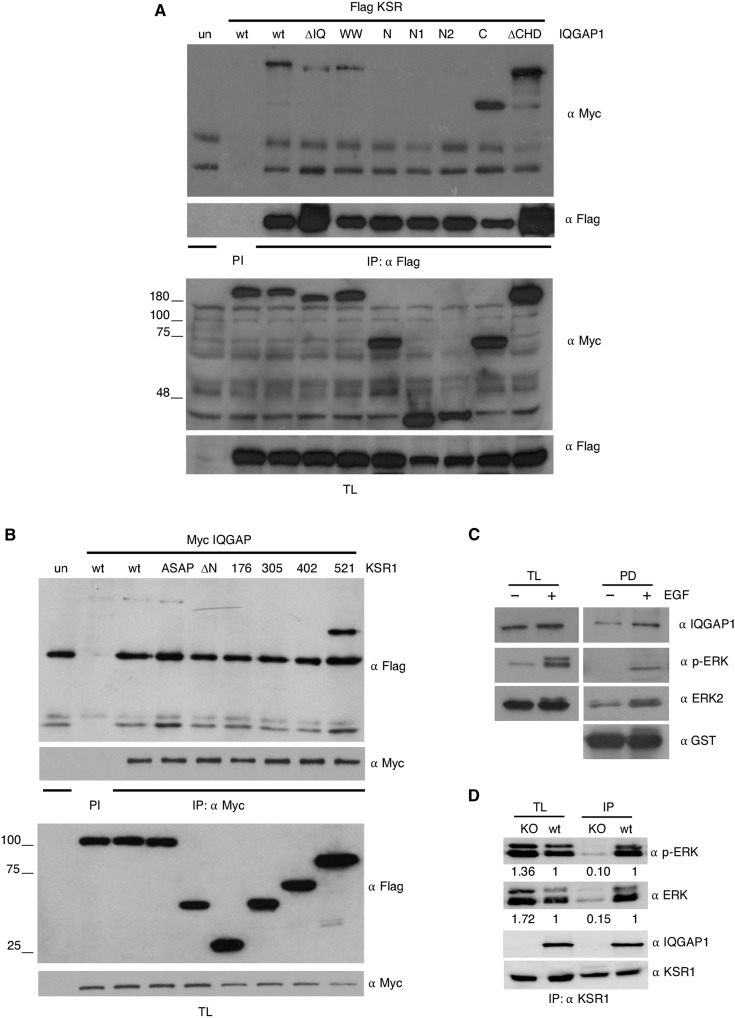
Identification of IQGAP1 and KSR1 binding sites. (**A**) The KSR1-binding domain is located in the C-terminal region of IQGAP1. HEK293T cells were transfected with the indicated Myc-tagged IQGAP1 constructs and Flag-tagged KSR1 (1.5 μg) and stimulated with EGF (50 ng/ml, 5 min). After 18-hour starvation, cells were stimulated with EGF (50 ng/ml, 5 min). The IQGAP1 constructs associated with KSR1 were determined by anti-Flag immunoprecipitation with subsequent anti-Myc immunoblotting. PI, immunoprecipitation using preimmune serum. (**B**) The IQGAP1-binding domain is located between amino acids 402 and 521 of KSR1. As in (A), cells were transfected with the indicated Flag-tagged KSR1 constructs and Myc-tagged IQGAP1. (**C**) ERK and IQGAP1 bind to the same region of KSR1. Lysates from HEK293T cells starved (−) or EGF-stimulated (+) were pulled down using GST-KSR1 301-600. Associated proteins were revealed by immunoblotting. (**D**) Effects of IQGAP1 down-regulation on KSR1-bound phosphorylated ERK, as determined by coimmunoprecipitation upon anti-KSR1 immunoprecipitation from wt and IQGAP1 CRISPR-Cas9–knockout (KO) SKMEL2 cells. Figures show signal intensity relative to the levels found in wt cells. All the results shown are representative of three to five independent experiments (see also fig. S2).

To identify the region(s) whereby IQGAP1 associated with KSR1, we used a series of deletion mutants for the different domains present in the protein (fig. S3A), and using coimmunoprecipitation assays, we determined which of these constructs were capable of binding to KSR1. It was found that all those constructs that contained the C-terminal region of IQGAP1, comprising amino acids 864 to 1657, which includes the GRD and the RGCT domains, could bind to KSR1. By contrast, the constructs lacking this region could not (Fig. 3A).

By the same strategy, we sought to characterize the KSR1 region(s) responsible for the interaction with IQGAP1. Once again, we used a battery of KSR1 deletion mutants covering the whole length of the protein (fig. S3B). Coimmunoprecipitation analyses showed that KSR1 associated with IQGAP1 through the CA4 region, spanning amino acids 402 to 521. However, the ERK-binding FXFP domain, included within this region, was not involved in this process, as the ASAP mutant (Phe > Ala substitution in the FXFP domain) retained its ability to bind IQGAP1 (Fig. 3B). Noticeably, the KSR1 construct 1 to 521, which lacks the CA5 region, bound to IQGAP1 with greater efficiency than the wild type and the ASAP constructs, which contain the CA5 region. This could imply that the CA5 region could, to some extent, attenuate binding to IQGAP1 (Fig. 3B). These results indicated that, in KSR1, the region involved in IQGAP1 binding was close to the ERK binding site, the FXFP domain (*15*). To verify this observation, we performed pull-down assays using a glutathione *S*-transferase (GST)–tagged KSR1 fragment spanning amino acids 301 to 600, which corroborated that this region harbored both IQGAP1- and ERK-binding determinants and that both proteins associated with KSR1 more prominently under EGF stimulation (Fig. 3C).

To determine whether IQGAP binding to KSR1 entailed some regulatory activity, we analyzed the levels of phosphorylated ERK bound to KSR1 in proliferating SKMEL2 cells, both wild-type and IQGAP1 knockout cells. IQGAP1 down-regulation resulted in a substantial reduction in the amount of phosphorylated ERK bound to endogenous KSR1 (Fig. 3D), suggesting that IQGAP1 could participate in the mechanism by which KSR1 functions as an ERK scaffold protein. Overall, these data clearly demonstrated that KSR1 and IQGAP1 can directly associate with each other, both in vivo and in vitro, with functional consequences.

### IQGAP1 participates in KSR1 C809Y trans-phosphorylation

**Fig. 4.**
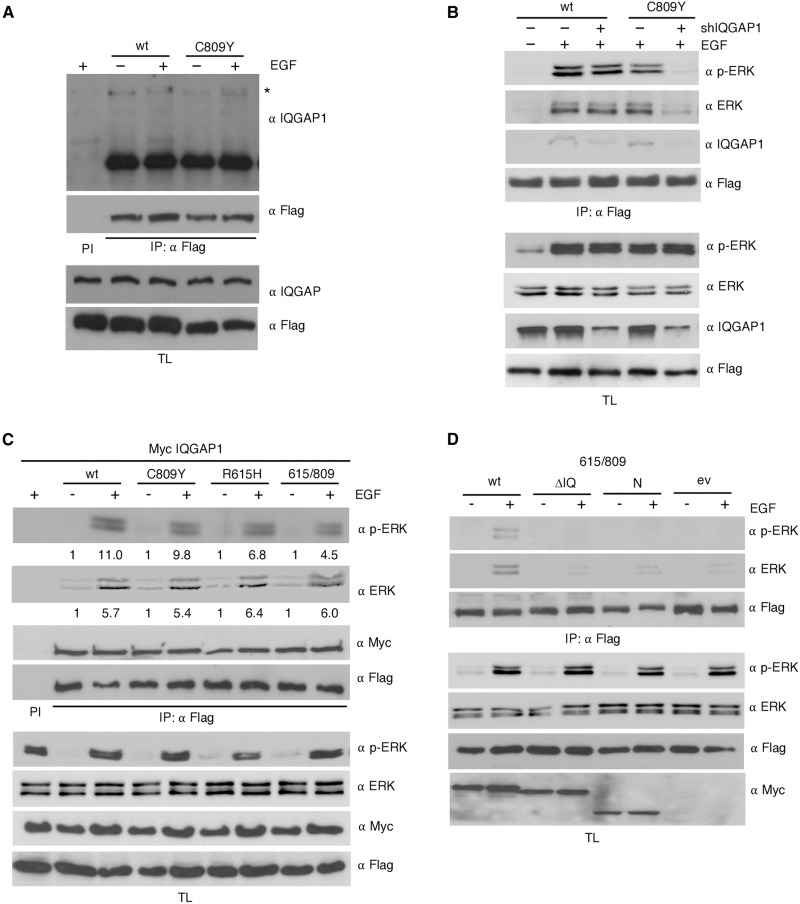
IQGAP1 participates in KSR1 trans-phosphorylation. (**A**) IQGAP1 interacts with KSR1 C809Y. Coimmunoprecipitation assay from HEK293T cells cotransfected with the indicated Flag-tagged KSR1 constructs (1.5 μg each), upon EGF stimulation (50 ng/ml, 5 min) where indicated (+), after 18-hour starvation (−). (**B**) Effects of IQGAP1 depletion on phosphorylated ERK binding to KSR1 C809Y. Coimmunoprecipitation assay in KSR1^−/−^ MEFs expressing the indicated Flag-tagged KSR1 constructs and transfected with shRNA against IQGAP1 where indicated (+) (1 μg), following EGF stimulation (+). (**C**) IQGAP1 overexpression facilitates KSR1 incorporation of phosphorylated ERK. Coimmunoprecipitation from HEK293T cells transfected with the indicated Flag-tagged KSR1 constructs plus Myc-tagged IQGAP1, in starved cells (−) or upon EGF stimulation where indicated (+). Figures show signal intensity relative to the levels in starved cells. (**D**) KSR1 incorporation of phosphorylated ERK mediated by IQGAP1 KSR-binding and MEK-binding mutants. Coimmunoprecipitation from HEK293T cells transfected with both Flag-tagged KSR1 615/809 and the indicated Myc-tagged IQGAP1 mutants (1 μg each), in starved cells (−) or upon EGF stimulation as shown (+); ev, empty vector. In all cases, immunoprecipitations were performed with a specific antibody (IP) or with preimmune serum (PI). All the results shown are representative of three to five independent experiments (see also fig. S3).

In light of these data, we determined whether IQGAP1 could compensate for KSR1 MEK–binding deficiency via trans-phosphorylation of KSR1-associated ERK. We observed that IQGAP1 coimmunoprecipitated with both wild type and C809Y KSR1, more prominently under EGF stimulation (Fig. 4A). To verify that IQGAP1 was, indeed, the scaffold responsible for complementing C809Y activity, in KSR1^−/−^ MEFs, we down-regulated IQGAP1 levels using short hairpin RNA (shRNA) and analyzed its impact on the ability of KSR1 to incorporate phosphorylated ERK. IQGAP1 depletion had no effect on the amount of phosphorylated ERK bound to wild-type KSR1 (Fig. 4B), probably because ERK would be cis-phosphorylated by KSR1-docked MEK, rendering IQGAP1 unnecessary. By contrast, IQGAP1 down-regulation markedly reduced the levels of phospho-ERK associated with the C809Y mutant (Fig. 4B).

We wanted to understand why, as shown in Fig. 1D, the KSR1 double mutant R615H/C809Y did not incorporate phosphorylated ERK in HEK293T cells, despite these cells expressing IQGAP1 and that such mutations lay outside the KSR1 IQGAP1–binding region. We hypothesized that in these cells, IQGAP1 levels might not be high enough to complement KSR1, and that a dimerizing wild-type KSR molecule could trans-activate C809Y, whereas cis-phosphorylation would account for the signal through the nondimerizing R615H mutant, neither case applying to R615H/C809Y, impaired both for homodimerization and for cis-activation. Therefore, we repeated the experiment in the presence of overexpressed IQGAP1. High IQGAP1 expression markedly augmented the amount of phosphorylated ERK present in the KSR1 dimerization–deficient mutant R615H and completely restored the ability of R615H/C809Y to incorporate phosphorylated ERK up to levels similar to those found in the wild-type protein (Fig. 4C).

To further substantiate this observation, we tested the capacity for rescuing ERK phosphorylation in KSR1 R615H/C809Y of several IQGAP1 mutants defective for features that we deemed necessary for trans-activation. While wild-type IQGAP1 could elicit incorporation of phosphorylated ERK onto R615H/C809Y, neither the N-terminal half of IQGAP1, lacking the KSR1-binding region, nor IQGAP1 ΔIQ, lacking the MEK binding site (fig. S3A), was capable of promoting phosphorylated ERK recruitment onto R615H/C809Y (Fig. 4D). Overall, these results demonstrated that IQGAP1 can support trans-phosphorylation of KSR1-bound ERK, provided that it can physically associate with KSR1 and that it can bind MEK.

### Both cis- and trans-phosphorylation contribute to KSR1-mediated ERK activation

**Fig. 5.**
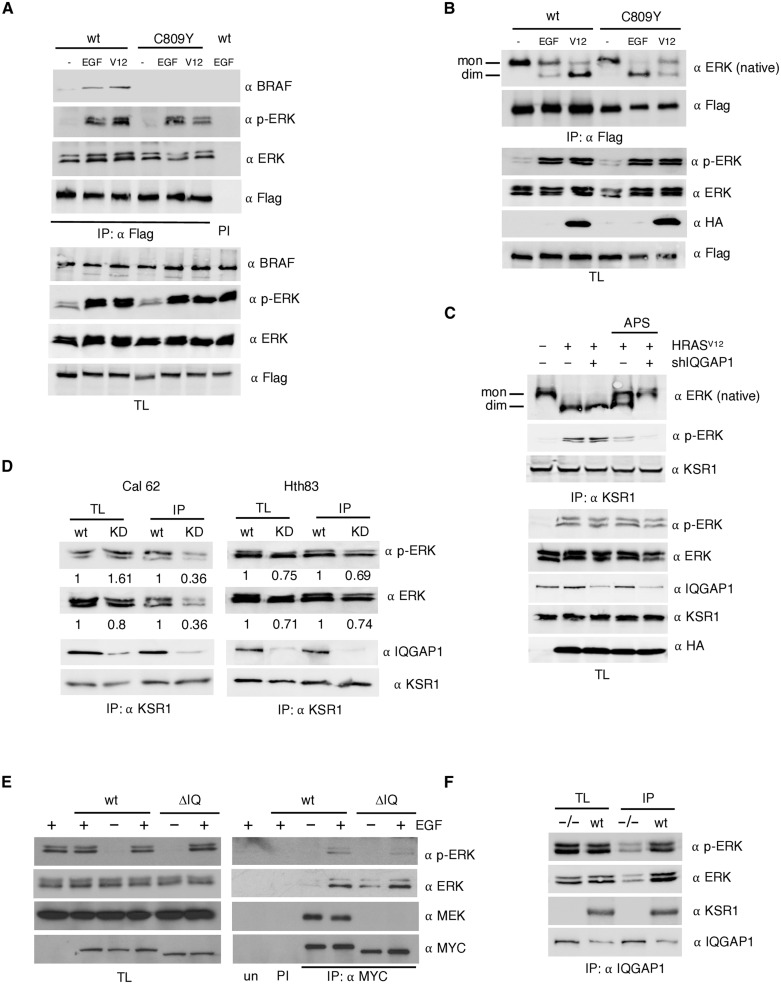
Cis- and trans-phosphorylation contribution to KSR1-mediated ERK activation. (**A**) The role of trans-phosphorylation on BRAF/KSR1 interaction. Coimmunoprecipitation assay from HEK293T cells cotransfected with the indicated Flag-tagged KSR1 constructs (1.5 μg each), upon EGF stimulation (50 ng/ml, 5 min), or when cotransfected with HRAS^V12^ (1 μg), after 18-hour starvation (−). (**B**) Impact of trans-phosphorylation on KSR1-mediated ERK dimerization. Performed as in (A), ERK dimerization was determined by native electrophoresis. Bands corresponding to ERK monomers and dimers are indicated. (**C**) ERK dimerization in response to KSR1 inactivation and/or IQGAP1 depletion. Determined in endogenous KSR1 immunoprecipitates from cells transfected with HRAS^V12^ (+) in the presence (+) or absence (−) of an shRNA against IQGAP1 after 18-hour starvation. Where indicated, cells were treated with APS-2-79 (5 μM, 2 hours). (**D**) Effects of IQGAP1 down-regulation on KSR1-bound phosphorylated ERK, as determined by coimmunoprecipitation upon anti-KSR1 immunoprecipitation from wt and IQGAP1 shRNA down-regulated (KD) Cal62 and Hth83 cells. Figures show signal intensity relative to the levels in wt cells. (**E**) ERK transphosphorylation in IQGAP1 MEK–binding mutant. HEK293T cells were transfected with the indicated MYC-tagged IQGAP1 constructs and stimulated with EGF where shown (+). (**F**) Effects of KSR1 down-regulation on IQGAP1-bound phosphorylated ERK, as determined by coimmunoprecipitation upon anti-IQGAP1 immunoprecipitation from wt and KSR1 knock-out (^−/−^) MEFs. In all cases, immunoprecipitations were performed with a specific antibody (IP) or with preimmune serum (PI). All the results shown are representative of three independent experiments.

It has been demonstrated that upon stimulation, KSR1 can heterodimerize with BRAF, thereby stimulating its catalytic activity (*26*). Thus, we determined whether, analogous to ERK activation, trans-phosphorylation could also elicit an interaction with BRAF. We observed that, whereas stimulation with EGF or by the ectopic expression of HRAS^V12^ markedly evoked BRAF association with wild-type KSR1, this was not the case for the C809Y mutant (Fig. 5A), indicating that trans-phosphorylation cannot promote KSR1 association to BRAF in the absence of MEK binding.

We previously demonstrated that scaffold proteins, in particular KSR, play an important role on ERK dimerization following its phosphorylation (*18*). Therefore, we investigated whether trans-phosphorylation could support ERK dimerization. To evaluate this, we tested the association of ERK dimers with KSR1 in response to two different incoming signals: EGF and the presence of ectopic HRAS^V12^. Noticeably, both stimuli readily induced ERK dimerization. However, in wild-type KSR1, ERK dimerization was promoted more profusely by HRAS^V12^, well known to induce a potent KSR1-mediated ERK activation (*9*). Conversely, in the C809Y mutant, ERK dimerized more prominently in response to EGF stimulation, known to signal through IQGAP1 for activating the RAS-ERK pathway (Fig. 5B) (*15*, *27*). These results could be interpreted as ERK cis-phosphorylation being the prevailing mode for inducing KSR-mediated ERK dimerization in response to RAS signals, while trans-phosphorylation is the main promoter of EGF-induced dimerization.

To evaluate the relative contribution of cis- versus trans-phosphorylation to KSR1-mediated ERK dimerization, we analyzed in further detail HRAS^V12^ signaling to endogenous KSR1. HRAS^V12^-induced ERK dimerization was unaffected by IQGAP1 knockdown (Fig. 5C), again advocating for cis-phosphorylation being the prevailing mechanism for HRAS^V12^-induced ERK dimerization via KSR1. On the other hand, treatment with the inhibitor APS-2-79 that binds to and stabilizes KSR1/2 in an inactive conformation (*12*) resulted in a marked reduction of HRAS^V12^-induced ERK dimerization, which was completely abolished by the concomitant depletion of IQGAP1 (Fig. 5C). These results suggested that when cis-phosphorylation through KSR1 is prevented, HRAS^V12^ can evoke IQGAP1-mediated trans-phosphorylation to bring about ERK dimerization.

Because cis- and trans-phosphorylation can contribute to different extents to KSR1-mediated ERK activation depending on the stimulus, we then determined whether such variability is also cell type dependent. In Fig. 3D, we showed that IQGAP1 depletion reduced phosphorylated ERK levels bound to KSR1 by about 90%. In Cal62 cells, it was found that such reduction exceeded 60%, whereas in Hth83 cells, IQGAP1 depletion decreased phosphorylated ERK levels only by around 30% (Fig. 5D). Overall, these results demonstrated that trans-phosphorylation contribute to KSR1-mediated ERK activation to different extents depending on the stimulus and the cellular context.

It was important to learn whether trans-phosphorylation was restricted to KSR1 or if other scaffold proteins were also subject to it. To test this, we analyzed whether MEK-binding–deficient IQGAP1 ΔIQ could incorporate phosphorylated ERK. As shown in Fig. 5E, in response to EGF stimulation, phosphorylated ERK coimmunoprecipitated with IQGAP1 ΔIQ at levels comparable to those found in the wild-type scaffold. To determine whether KSR1 could be the scaffold responsible for IQGAP1 trans-phosphorylation, we analyzed the levels of phosphorylated ERK bound to IQGAP1 in proliferating wild-type and KSR1-knockout MEFs. Noticeably, KSR1 down-regulation reduced the amount of phosphorylated ERK bound to endogenous IQGAP1 (Fig. 5F), showing that KSR1 can regulate the role of IQGAP1 as an ERK scaffold protein. Together, these results demonstrated that trans-phosphorylation is not unique to KSR1 and that it can be a widespread phenomenon among ERK scaffold proteins.

### Trans-phosphorylation is a factor in KSR1-mediated cellular events

**Fig. 6.**
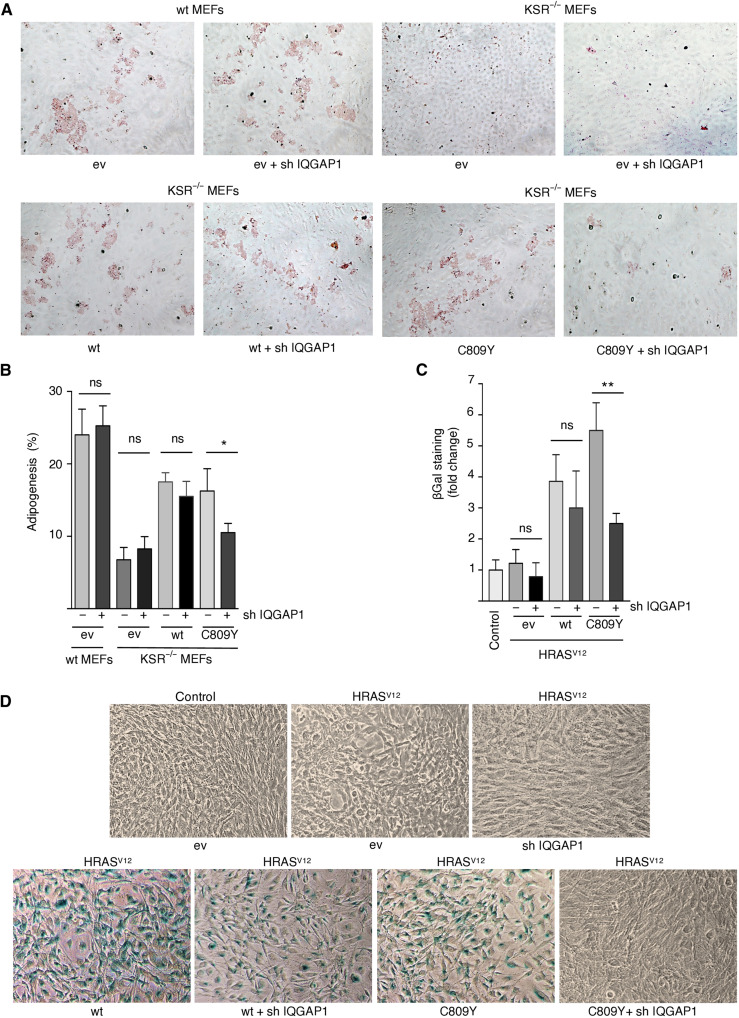
Trans-phosphorylation role in KSR1-mediated cellular processes. (**A**) The role of IQGAP1 in adipogenesis mediated by KSR1. Micrographs of KSR1 wt and KSR1^−/−^ MEFs transfected with the indicated KSR1 constructs in the presence of an shRNA against IQGAP1 (1 μg each) where indicated, after 8 days of treatment with MDI. Adipogenesis was revealed by oil-red staining. (**B**) Quantification of the above results. Data show means ± SEM of three independent experiments. *P* values: *P < 0.05; ns, not significant, by Student’s *t* test. (**C**) The role of IQGAP1 in RAS-induced senescence mediated by KSR1. KSR1^−/−^ MEFs were transfected with HRAS^V12^ and the indicated KSR1 constructs in the presence (+) or absence (−) of an shRNA against IQGAP1. Senescence was scored by β-galactosidase staining. Data show means ± SEM of three independent experiments, relative to the value in cells not transfected with RAS (control). *P* values: **P < 0.01; ns, not significant, by Student’s *t* test. (**D**) Micrographs of KSR1^−/−^ MEFs transfected with oncogenic RAS and the indicated KSR1 constructs in the presence of shRNA against IQGAP1 where shown (1 μg each). Senescence was revealed by β-galactosidase staining. All the results shown are representative of three to five independent experiments.

It was essential to gain some insight into the biological significance of trans-phosphorylation. It is well documented that the RAS-ERK pathway is deeply implicated in the regulation of cell differentiation (*28*). For example, KSR1 has been shown to regulate adipogenic differentiation (*29*). Wild-type MEFs treated with a cocktail of insulin, dexamethasone, and methylisobutylxanthine (MDI) undergo adipocytic differentiation, as demonstrated by the accumulation of triglyceride droplets. Such process is impaired in KSR1^−/−^ MEFs but can be rescued by ectopic expression of wild-type KSR1 (*29*). We observed that the expression of C809Y was capable of restoring adipogenesis as efficiently as wild-type KSR1 (Fig. 6A). However, when IQGAP1 was down-regulated by an shRNA, while unaffecting adipogenesis in wild-type MEFs and in KSR1^−/−^ MEFs expressing wild-type KSR1, it substantially diminished C809Y capacity for reestablishing adipocytic differentiation in the latter (Fig. 6, A and B). These results indicate that trans-phosphorylation via IQGAP1 has a role on the regulation of adipogenesis mediated by KSR1.

In the same vein, it has been shown that KSR1 C809Y can efficiently support ERK-dependent, HRAS^V12^-induced senescence in KSR1^−/−^ MEFs (*21*). Therefore, we examined whether IQGAP1 also participated in this process. shRNA-mediated IQGAP1 down-regulation did not affect HRAS^V12^-induced senescence as mediated by wild-type KSR1, but it significantly impaired the ability of C809Y to facilitate this process (Fig. 6, C and D). Overall, these results demonstrated that IQGAP1 plays an essential role in conveying ERK-activating signals through MEK-binding deficient KSR1 and that such trans-phosphorylation process can be a determinant in KSR1-mediated biological processes.

### Compensation of KSR1 and IQGAP1 levels in cancer samples

**Fig. 7.**
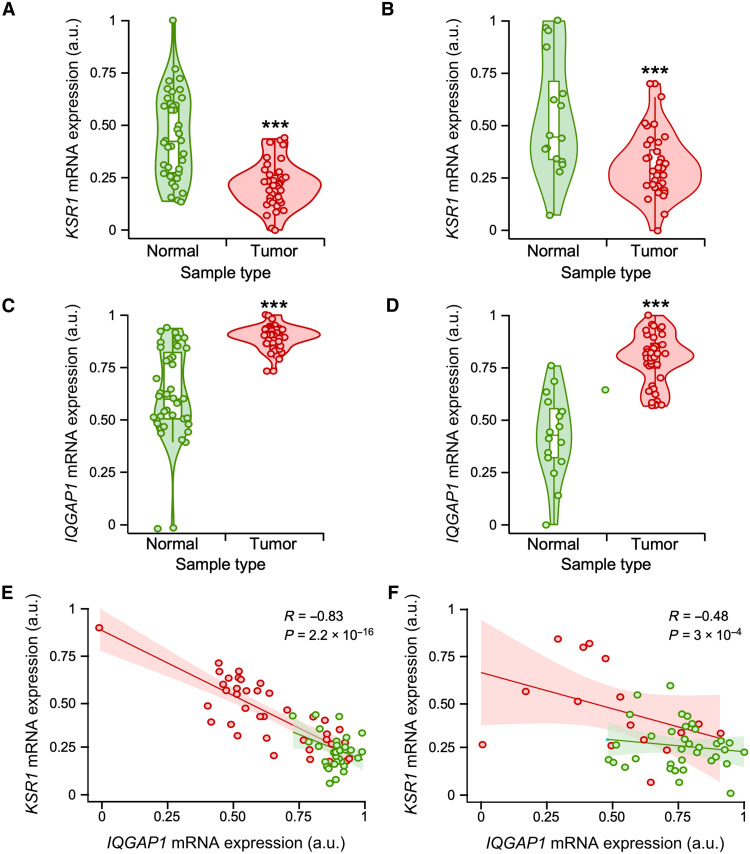
Negative correlation of *KSR1* and *IQGAP1* mRNA levels in tumors. (**A** to **D**) Relative expression levels of *KSR1* (A and B) and *IQGAP1* (C and D) transcripts in healthy and tumor samples obtained from the microarray datasets GSE15471 (A and C) and GSE16515 (B and D). (**E** and **F**) Regression graph showing the negative correlation between KSR1 and IQGAP1 transcripts levels in microarray datasets GSE15471 (E) and GSE16515 (F). Dots, violin plots, and regression line colors correspond to tumor (red) and healthy (green) samples from the indicated dataset. ***P < 0.001 (A to D). In (D) and (F), the regression coefficient and *P* value are indicated in each case (see also fig. S3).

The up-regulation of IQGAP1 in *KRS1*^−/−^ MEFs (Fig. 2B) suggested that there could be potential compensatory events between these scaffold molecules in cells. To further assess this concept, we analyzed the expression of both *KSR1* and *IQGAP1* transcripts in a number of publicly available transcriptomal datasets present in both the Pan-Cancer and Gene Expression Omnibus (GEO) repositories. The expression of *KSR1* and *IQGAP1* mRNAs was not significantly deregulated in most datasets analyzed (table S1). However, we found two GEO pancreatic adenocarcinoma datasets (GSE15471 and GSE16515) in which the *KSR1* (Fig. 7, A and B) and *IQGAP1* (Fig. 7, C and D) transcripts were down-regulated and up-regulated when compared to healthy tissue control, respectively. Consistent with this finding, coexpression analyses indicated an inverse correlation between the levels of these two transcripts in both datasets (Fig. 7, E and F). This negative correlation was observed regardless of the tumoral status of the samples (Fig. 7, E and F), suggesting that it might also apply to normal cells. Similar studies indicated that such negative correlation seems specific for *KSR1*, because *KSR2* mRNA does not show any type of differential expression in all the datasets surveyed (fig. S4, A and B). However, in the case of the IQGAP family, we found that IQGAP3, but not IQGAP2, displayed an IQGAP1-like behavior in both the GSE15471 and GSE16515 datasets (fig. S4, A and B). Collectively, these data suggest that changes in expression in one of these scaffolds can be counterbalanced by opposite changes in some of the other scaffold counterparts. It is worth noting, however, that the expression of transcripts for KSR and IQGAP family members are not usually deregulated in cancer cells when compared to healthy tissues in most gene expression datasets (table S1).

### Trans-phosphorylation weakens KSR inhibitor efficacy

**Fig. 8.**
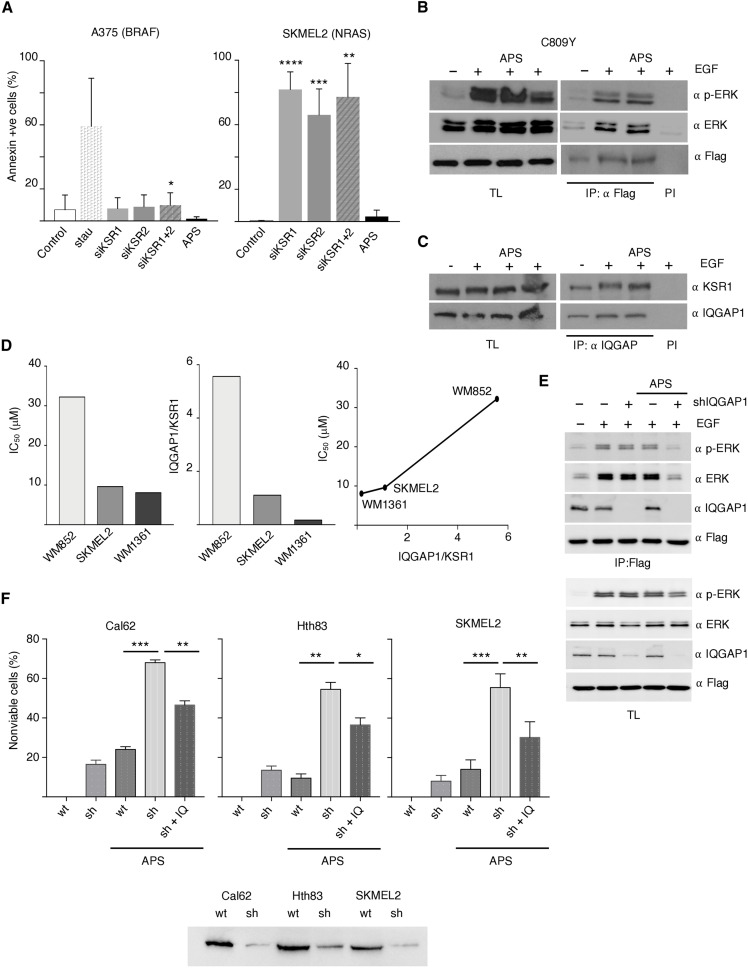
IQGAP1 impact on APS 2-79 efficacy. (**A**) APS-2-79 does not phenocopy the effect of KSR1/2 ablation. A375 and SKMEL2 cells were transfected with the indicated shRNAs or an empty vector as a negative control (c) or treated with APS-2-79 (5 μM, 48 hours). Staurosporine (0.5 μM, 48 hours) was used as a positive control. Apoptosis was evaluated 48 hours after transfection by scoring annexin V levels. Data show means ± SEM from three independent experiments. *P* values: ****P < 0.001, ***P < 0.005, **P < 0.01, and *P < 0.05 by Student’s *t* test. (**B**) APS-2-79 does not affect phosphorylated ERK levels incorporated to KSR1. Endogenous levels bound to KSR1 C809Y were determined by coimmunoprecipitation analyses in APS-2-79–treated cells, stimulated with EGF (50 ng/ml, 5 min) where shown (+) after 18-hour starvation. (**C**) APS-2-79 does not affect KSR1-IQGAP1 interaction. Association of the endogenous proteins was determined by coimmunoprecipitation analyses. (**D**) IC_50_ and IQGAP1/KSR1 ratio in the indicated NRAS-mutant cell lines and correlation between both parameters. Data show mean of three independent experiments. (**E**) Impact of IQGAP1 depletion on the APS-2-79 inhibitory effect on KSR1. As in (C), IQGAP1 was depleted by shRNA where indicated. (**F**) IQGAP1 depletion effects on APS-2-79 efficacy. The indicated tumor cells: wt, IQGAP1 down-regulated using shRNAs (sh), and IQGAP1 down-regulated transfected with an ectopic IQGAP1 (1 μg; sh + IQ) were treated with APS 2-79 where indicated. Data show means ± SEM, % of nonviable cells for three independent experiments. *P* values: ***P < 0.005, **P < 0.01, and *P < 0.05 by Student’s *t* test. Bottom: IQGAP1 levels in the indicated cell lines. In all cases, immunoprecipitations were performed with a specific antibody (IP) or with preimmune serum (PI). All the results shown are representative of three to five independent experiments (see also fig. S4).

The importance of KSR1 in the conveyance of RAS oncogenic signals (*13*) has placed it in the limelight as a therapeutic target. This has stimulated the development of small-molecule inhibitors such as APS-2-79. However, as monotherapy, APS-2-79 has only modest cytotoxic effects, restricted to RAS-mutant cell lines (*12*). In agreement, we found that APS-2-79 hardly affected cell viability in NRAS-mutant cell lines (fig. S5A). However, unlike BRAF-mutant cells, small interfering RNA (siRNA)–mediated KSR1/2 depletion in tumor cells harboring oncogenic RAS induced a potent apoptotic response (Fig. 8A), indicating that APS-2-79 was not efficiently inhibiting KSR activity. Thus, we determined whether KSR trans-phosphorylation could underlie the poor efficacy of APS-2-79. We observed that APS-2-79 treatment could not prevent incorporation of phosphorylated ERK on KSR1 C809Y (Fig. 8B), nor did it affect KSR1-IQGAP1 interaction in EGF-stimulated cells (Fig. 8C), demonstrating that APS-2-79 would not inhibit KSR1 trans-phosphorylation mediated by IQGAP1. Moreover, when we evaluated APS-2-79 half-maximal inhibitory concentration (IC_50_) in a series of NRAS-mutant cell lines (fig. S5B), we observed that it correlated with the IQGAP1/KSR1 ratio, in such a way that those cells harboring the highest IQGAP1 levels relative to those of KSR1 displayed the greatest resistance to APS-2-79 (Fig. 8D).

The above results suggested that IQGAP1 could be facilitating KSR-bound ERK phosphorylation, bypassing APS-2-79 inhibitory effect on KSR-associated CRAF and MEK activation. To test this hypothesis, we analyzed the impact of IQGAP1 depletion on APS-2-79 effectiveness. It was found that the absence of IQGAP1 potentiated the ability of APS-2-79 to inhibit KSR-associated ERK phosphorylation (Fig. 8E). Furthermore, when we tested the effect of APS-2-79 on a series of RAS-mutant cell lines in which IQGAP1 expression was down-regulated via shRNAs, we observed that APS-2-79 cytotoxicity was significantly higher in cells with reduced IQGAP1 levels compared to the corresponding parental cells. Moreover, transfection of ectopic IQGAP1 into IQGAP1 down-regulated cells significantly rescued cellular viability in response to APS-2-79 treatment (Fig. 8F). Overall, these results demonstrate that IQGAP1 down-regulation improves the efficacy of APS-2-79 as a KSR1 inhibitor.

## DISCUSSION

While our knowledge of MAPK scaffold proteins has grown substantially over the past years, unexplained nuances are still common. The case of KSR1 C809Y mutant deficient for binding MEK being able to incorporate phosphorylated ERK (*21*) is one example. Here, we demonstrate that such an event is the consequence of a process that we have named trans-phosphorylation, in which kinase-substrate phosphorylation reactions occur across different scaffold protein complexes bound to each other.

It has been demonstrated that KSR can dimerize (*23*). Therefore, the finding that the KSR1 double mutant R615H/C809Y, deficient both for dimerization and for binding MEK, is incapable of incorporating phosphorylated ERK indicates that a trans-acting MEK bound to either a homodimerizing KSR1 or a heterodimerizing KSR2 could catalyze C809Y-bound ERK phosphorylation. The possibility of a free, cytoplasmic MEK being responsible for such process must be discarded, as ERK bound to the KSR1 double mutant should be phosphorylatable by soluble MEK. The occurrence of trans-acting processes has been previously described in the phosphorylation cascade mediated by KSR. As such, RAF interaction with KSR in cis triggers a conformational switch on MEK that facilitates its phosphorylation by another RAF molecule in trans (*30*). However, it is unknown whether in this case RAF is free or bound to another scaffold protein. Trans-phosphorylation appears to be a feature not unique to KSR1, because we demonstrate that MEK binding–deficient IQGAP1 is also subject to trans-phosphorylation.

More interestingly, we demonstrate that trans-phosphorylation can also occur between different scaffold protein species. We show that in a cellular environment lacking KSR1/2, IQGAP1 can support the trans-phosphorylation of KSR1 C809Y–bound ERK. For this, a direct association between IQGAP1 and KSR1 must take place. IQGAP1 and ERK bind to KSR1 in close proximity. Associations between different scaffold species are not unprecedented. In this respect, adaptor, docking, and scaffold proteins of different types have been reported to interact, forming “macro” signaling platforms (*31*). With respect to RAS-ERK pathway scaffolds, interactions have been demonstrated for MP1 and MORG1 (*32*), paxillin and GAB1 (*33*), and IQGAP1 with MP1 (*34*) and β-arrestin2 (*35*). However, how such interactions affect ERK signals is largely understudied.

Our observation that high IQGAP1 levels appear to compensate for such absence in KSR1/2-deficient cells is worth noting. We observed an analogous situation in several types of tumors, in which IQGAP1 and KSR1 levels fluctuate inversely, suggesting that changes in expression of one scaffold could be counterbalanced by opposite changes in the other scaffold, once again suggesting for a functional interconnectivity between different scaffold species.

Our data reveal the existence of a functional association between different scaffold proteins. This adds a higher-order, additional degree of complexity to the already highly complex regulation of signal flux through the RAS-ERK pathway, in which macromolecular assemblies play a substantial role (*36*, *37*). Because scaffold species display singular spatial selectivity (*3*) and distinctive affinities for defined pools of substrates (*10*), complexes made up of different scaffolds, competent for cooperating among themselves, may constitute a type of regulatory node whereby distinct, spatially defined, incoming signals are integrated, and outgoing signals are diversified with respect to substrate usage (*38*).

Furthermore, we demonstrate the cooperation of two different types of scaffolds in the regulation of a kinase cascade. Such cooperativity opens the possibility that scaffolds, missing one or more kinases, could associate in trans with other incomplete scaffold species to permit signal flux, allowing the complementation and compensation for each other’s deficiencies. As such, incomplete scaffold complexes, theoretically incompetent in their role, as is the case for our KSR1 and IQGAP1 MEK–binding mutants, would be fit for signaling, as we demonstrate. In this fashion, signal transmission could take place under circumstances where specific scaffolds would fail if acting on their own. Cooperation of this nature would be particularly beneficial under circumstances where the collaborating scaffolds display different affinities for a kinase whose concentration is limiting. In this respect, we demonstrate that trans-phosphorylation via IQGAP1 can make biological processes happen, such as adipocyte differentiation and cellular senescence, for which a deficient KSR1 activity would fail.

**Fig. 9.**
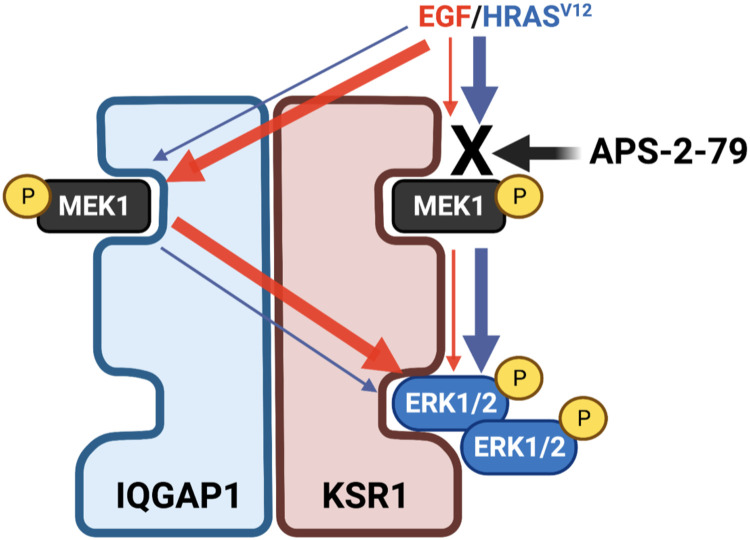
A model for cis- and trans-phosphorylation impact on ERK activation. As demonstrated in KSR1/IQGAP1 complexes in response to HRAS^V12^ signals or EGF stimulation. Trans-phosphorylation via IQGAP1 can bypass the inhibitory effect of APS-2-79 on KSR1-bound ERK activation.

We show that in HEK293T cells, which express endogenous IQGAP1, IQGAP1 does not efficiently support KSR1 trans-phosphorylation unless it is overexpressed. Because IQGAP1 has been shown to bind more than 50 different proteins (*39*), one possible explanation is that, in HEK293T, endogenous IQGAP1 is repressed for trans-phosphorylation by one of its partners and is only relieved when IQGAP1 levels exceed those of its putative repressor. In this respect, our results demonstrate that cis- and trans-phosphorylation contribute to different extents to KSR1-bound ERK activation depending on the cell type and on the incoming stimulus. As depicted in Fig. 9, cis-phosphorylation would be the principal mechanism for activating KSR1-bound ERK when evoked by oncogenic RAS signals, whereas IQGAP1-mediated trans-phosphorylation would be the prevailing means in response to EGF stimulation. This could constitute a safeguard mechanism for warranting signal flux through KSR1 regardless of the activating stimulus.

Consistent with this notion, we demonstrate that trans-phosphorylation across different scaffold species underlies the poor antineoplastic performance of a scaffold-directed small-molecule inhibitor. Having two alternative routes whereby signal flux can be driven through KSR1, it could be hypothesized that the system can somehow perceive the blockade of cis-phosphorylation, consequently unleashing IQGAP1-mediated trans-phosphorylation to prevent the blockade of KSR1 functions. Thus, the inhibitory effect of APS-2-79 on KSR-bound MEK activation (*12*) could be bypassed by IQGAP1-mediated trans-phosphorylation. Consistent with this hypothesis, we show that depletion of IQGAP1 boosts the capacity of APS-2-79 to inhibit KSR-bound ERK phosphorylation and its consequential cytotoxic effects. However, although improved, even in the absence of IQGAP1, APS-2-79 effects remain modest. One possible explanation is that other types of scaffold proteins may also be competent for trans-phosphorylating KSR. This would be consistent with our results showing that even in the absence of IQGAP1, the levels of KSR-bound phosphorylated ERK are still substantial. If this were the case, any scaffold-aimed therapeutic strategy based on inhibiting signal flux upstream of ERK would be bound to fail. Instead, only directly inhibiting scaffold-associated ERK activation, for example, by small molecules aimed at impeding ERK scaffold binding, would have some chance of success.

## MATERIALS AND METHODS

### Cell lines, drugs, and reagents

Cell lines were grown in a humidified incubator at 37°C and 5% CO_2_ in Dulbecco’s modified Eagle’s medium (DMEM; Thermo Fisher Scientific) supplemented with 10% fetal bovine serum (FBS; Gibco) and 1% penicillin-streptomycin (10,000 U/ml; Thermo Fisher Scientific). HEK293T [American Type Culture Collection (ATCC) catalog no. CRL-3216, RRID:CVCL_0063], HeLa (ATCC catalog no. CCL-2, RRID:CVCL_0030), A375 (ATCC catalog no. CRL-3224, RRID:CVCL_6233), 501-MEL (ATCC RRID:CVCL_4633), CJM (ATCC RRID:CVCL_U797), SKMEL2 (ATCC catalog no. HTB-68, RRID:CVCL_0069), SKMEL28 (CSL catalog no. 300337/p495_SK-MEL-28, RRID:CVCL_0526), MEL-JUSO (DSMZ CLS catalog no. 300282/NA, RRID:CVCL_1403), WM1351 (RRID:CVCL_0672), and WM852 (RRID:CVCL_0684) were purchased from Rockland Inc.; Cal62 (RRID:CVCL_1112) and Hth83 (RRID:CVCL_0046) were obtained from P. Santisteban (IIBM, Madrid); and wild-type and KSR1^−/−^ MEFs were a gift from J. Lozano (University of Málaga, Spain). EGF was obtained from Sigma-Aldrich (no. E9644), APS-2-79 was purchased from Med-Chem Express (no. HY-100627), Vemurafenib (PLX4032) was acquired from Selleck Chemicals (no. S1267), and Staurosporine was obtained from Sigma-Aldrich (no. S5921). Doxorubicin was purchased from Sigma-Aldrich (no. 25316-40-9).

### Gene knockdown and overexpression

shRNAs against human KSR1 (TRCN 006226, TRCN 006227, TRCN 006229, and TRCN 006230 XM 290793), human KSR2 (TRCN 007062, TRCN 335901, TRCN 199619, TRCN 199136, and TRCN 195374 NM 173593), and human IQGAP1 (TRCN 47485, TRCN 47487, TRCN 298928, TRCN 298930, and TRCN 298931) were obtained from Sigma-Aldrich. siRNA against KSR1 (no. sc-35762) and siRNA against IQGAP1 (no. sc-35700) were purchased from Santa Cruz Biotechnology. siRNAs and shRNAs were transfected with Lipofectamine RNAiMAX Transfection Reagent (Thermo Fisher Scientific, no. 13778150) following the manufacturer’s directions.

pCDNA3 MYC IQGAP1 WW, ∆IQ, ∆CHD, N1, N2, N, and C were generated by D. Sacks; pEF-BOS MYC IQGAP1 was supplied by K. Kaibuchi; pCDNA3 Ksr1 GLU was provided by W. J. Fantl; pHis parallel KSR1, pCMV FLAG KSR1 wild type, C809Y, ASAP, 176, 305, 402, 521, and ∆N were a gift from J. Lozano. pCEFL HA HRAS^V12^ has been previously described (*40*).

We introduced the mutation R615H into KSR1 wild type and C809Y by site-directed mutagenesis (QuikChange II site-directed mutagenesis kit, Agilent, no. 200523) using the following primers: forward, 5′GAACTACCGGCAGACGCATCATGAGAACGTGGTGC3′ and reverse, 5′GCACCACGTTCTCATGATGCGTCTGCCGGTAGTTC3′. These constructs were subcloned pCEFL by polymerase chain reaction using the primers KSR1 Not I forward: 5′TGCTTCGCGGCCGCCTACATCTTTGGATTACC3′ and Eco RI Flag KSR1 forward: 5′GGTGGTGAATTCATGGACTACAAGGACGAT3′.

HEK293T cells were transfected with polyethylenimine (PEI) (1 mg/ml) in a 1:3 (DNA:PEI) ratio. HeLa cells were transfected using Lipofectamine LTX (Invitrogen, Thermo Fisher Scientific, no. 15338100) and SKMEL-2 melanoma cells were transfected with Lipofectamine 3000 (Invitrogen, Thermo Fisher Scientific, no. L3000015) as specified by the manufacturers.

Mouse embryonic fibroblasts (MEFs) were transfected by nucleofection. Around 8 × 10^6^ cells were washed with 1× phosphate-buffered saline (PBS) and trypsinized. They were centrifuged at 1500 rpm for 5 min, and the pellet was resuspended in 400 μl of electroporation solution (Ingenio Electroporation Kit, Mirus, no. MIR50117) with 3 μg of the corresponding DNAs. The cells in suspension were electroporated by an electrical pulse; duration and voltage were as detailed by the MEF-specific program (A 023) in the nucleofector (Lonza).

### Western blot analyses

Cell plates were collected on ice, the culture medium was removed, and the cells were washed in cold 1× PBS and harvested in 200 to 500 μl of lysis buffer [20 mM Hepes (pH 7.5), 10 mM EGTA, 40 mM β-glycerophosphate, 1% NP-40, 2.5 mM MgCl_2_, 1 mM NaVO_4_, 1 mM dithiothreitol (DTT), and protease inhibitors: aprotinin (10 μg/ml) and leupeptin (10 μg/ml)]. Cell lysates were cleared at 13,000 rpm for 10 min at 4°C, and protein concentration was quantified using the Bradford method at 620 nm; 5× Laemmli loading buffer was added to samples of 30 μg of protein, and the mix was boiled at 95°C for 5 min.

Proteins were resolved by SDS–polyacrylamide gel electrophoresis (PAGE). Native gel protein electrophoresis was performed as previously described (*20*). Gels were transferred to nitrocellulose membranes (AmershamProtran Supported 0.45 NC, GE Healthcare Life Sciences). Membranes were blocked in tris-buffered saline–Tween (TBS-T) containing 4% bovine serum albumin (BSA; blocking solution). Blots were incubated from 1 hour at room temperature to overnight at 4°C (depending on the antibody performance) with the different antibodies prepared in blocking solution. Subsequently, the blots were incubated for 1 hour with shaking at room temperature with anti-rabbit immunoglobulin (Ig; Bio-Rad, no. 170-5046) or anti-mouse Ig (Bio-Rad, no. 170-5047) secondary antibodies conjugated with peroxidase (1:10,000) in 2% milk (GE Healthcare) TBS-T. Proteins were detected by chemiluminescence with an enhanced chemiluminescent system and autoradiography (Konica films).

### Coimmunoprecipitation assays

Cell lysates were centrifuged at 13,000 rpm at 4°C for 10 min. The cleared lysates were quantified and 30 μg of protein from the total lysate was separated and 5× loading buffer Laemmli was added. The antibody (0.5 to 1 μg) specific for immunoprecipitation was added to 300 μg of protein and incubated with rocking at 4°C from 2 hours to overnight. Twenty microliters of Protein G–Sepharose 4B (GE Healthcare, no. 17-0756-01) was added and incubated for 20 min at 4°C shaking. The immunocomplexes were precipitated by centrifugation. Beads were washed once with lysis buffer and twice with cold 1× PBS; 1% NP-40. Last, the beads were resuspended in 20 μl of 2.5× loading buffer Laemmli and boiled 5 min at 95°C and then analyzed by SDS-PAGE as previously described. Coimmunoprecipitations were repeated at least three times in independent experiments.

### Antibodies

The following antibodies were used: mouse monoclonal anti-Flag M2 (Sigma-Aldrich, catalog no. F1804, RRID:AB_262044); rabbit polyclonal Anti-Glu-Glu-epitope Tag (Millipore, catalog no. AB3788, RRID:AB_91589); rabbit monoclonal anti-p44/42 MAPK (Erk1/2; 137F5) (Cell Signaling Technology, catalog no. 4695, RRID:AB_390779); mouse monoclonal anti-p-ERK (E-4) (Santa Cruz Biotechnology, catalog no. sc-7383, RRID:AB_627545); mouse monoclonal anti-MAP Kinase, Activated (Diphosphorylated ERK-1&2) (Sigma-Aldrich, catalog no. M9692, RRID:AB_260729); mouse monoclonal anti-MAP Kinase, Activated (Diphosphorylated ERK-1&2) (Sigma-Aldrich, catalog no. M8159, RRID:AB_477245); rabbit monoclonal anti-Phospho-MEK1/2 (Ser^217^/Ser^221^; 41G9) (Cell Signaling Technology, catalog no. 9154, RRID:AB_2138017); rabbit monoclonal anti-MEK1/2 (D1A5) (Cell Signaling Technology, catalog no. 8727, RRID:AB_10829473); mouse monoclonal anti-alpha-Tubulin (Sigma-Aldrich, catalog no. T5168, RRID:AB_477579); rabbit polyclonal Anti-Myc Tag antibody (Millipore, catalog no. 06-549, RRID:AB_310165); mouse monoclonal anti-c-Myc (9E10) (Thermo Fisher Scientific, catalog no. MA1-980, RRID:AB_558470); mouse monoclonal anti-Ksr-1 (E-5) (Santa Cruz Biotechnology, catalog no. sc-515924); rabbit monoclonal anti-KSR1 [EPR2421Y] (Abcam, catalog no. ab68483, RRID:AB_11157290); mouse monoclonal Anti-IQGAP1 (C-24) (BD Biosciences, catalog no. 610611, RRID:AB_397945); mouse monoclonal Anti-IQGAP1 (C-9) (Santa Cruz Biotechnology, catalog no. sc-376021, RRID:AB_10988556); Immun-Star Goat Anti-Mouse (GAM)–horseradish peroxidase (HRP) Conjugate antibody (Bio-Rad, catalog no. 170-5047, RRID:AB_11125753); Immun-Star Goat Anti-Rabbit (GAR)–HRP Conjugate antibody (Bio-Rad, catalog no. 170-5046, RRID:AB_11125757); goat anti-Rabbit IgG (H + L) Highly Cross-Adsorbed Secondary Antibody, Alexa Fluor 488 (Thermo Fisher Scientific, catalog no. A-11034, RRID:AB_2576217); and goat anti-Mouse IgG (H + L) Highly Cross-Adsorbed Secondary Antibody, Alexa Fluor 594 (Thermo Fisher Scientific, catalog no. A-11032, RRID:AB_2534091).

### Proximity ligation assays

Transfected HeLa cells were grown to subconfluence in coverslips (10 mm Ø), washed with 1× PBS, and fixed with 4% paraformaldehyde in 1× PBS for 10 min at room temperature. Later, they were washed twice with 1× PBS for 5 min, followed by one wash with 0.1 M glycine and two washes with 1× PBS. Subsequently, they were permeabilized for 10 min with 0.1 M glycine and 0.5% Triton X-100 in PBS, followed by three washes with 1× PBS for 5 min. Then, the cells were blocked for 15 min by adding one drop over each glass of 3% BSA and 0.01% Triton X-100 in 1× PBS. The primary antibodies were prepared in blocking solution in a dilution from 1:75 to 1:200 depending on the antibody specificity; they were also added as a drop over the glass and incubated for 1 hour in a humid chamber. MINUS and PLUS PLA probe solution was prepared in a 1:3 ratio with blocking buffer and the mix was incubated for 20 min at room temperature. After the primary antibody incubation, the cells were washed twice for 5 min with buffer A [0.15 M NaCl, 0.01 M tris base, 0.05% Tween 20 (pH 7.4) filtered] and then a drop of PLA probe solution was added per glass and incubated at 37°C for 1 hour. During incubation, the ligation solution (ligation buffer diluted 1:5 and ligase 1:40 in ultrapure water) was prepared, adding the ligase just before use. One drop of ligation solution was added over the glasses after two 5-min washes with buffer A, and it was incubated at 37°C for 30 min. After ligation, the cells were washed twice with buffer A, followed by the addition of the amplification solution (amplification buffer diluted 1:5 and polymerase 1:80 in ultrapure water). The amplification step lasts 100 min at 37°C. After the incubation period, the cells were washed twice for 10 min with buffer B (0.1 M NaCl, 0.2 M tris base, and tris-HCl, pH 7.5), and they were left at 4°C overnight. The day after, a secondary antibody (conjugated with a fluorophore) specific for the primary antibody was added for 1 hour in the humidity chamber and washed twice with 1× PBS. Last, the glasses were set over a slide in mounting media with 4′,6-diamidino-2-phenylindole and sealed with clear nail polish. Cells were examined by fluorescence microscopy (photomicroscope Axiophot, Carl Zeiss). Images were processed using ImageJ software.

### cPLA2 activation assays

KSR1^−/−^ MEFs were nucleofected, as previously described, with the different KSR1 constructions. Forty-eight hours after transfection, the cells were deprived of serum, and H^3^ arachidonic acid (1 μCi/ml; PerkinElmer no. NET298Z05) was added to the medium. After 18 hours of incubation, the cells were washed twice with filtered fatty acid–free DMEM, 5 mM Hepes (pH 7.5), and 0.2% BSA, the medium was replaced by this, and EGF was added (50 ng/ml, 2 hours) where applicable. Two hours later, 500 μl of the medium was taken and mixed with 2 ml of scintillation liquid in a counting vial, and emission was measured in a scintillation counter.

### Apoptosis analyses

One million cells were plated per T6 plate well. Twenty-four hours later, the cells were transfected with the corresponding shRNAs, and treatments were added as necessary. In parallel, the same transfections were carried out in P60 plates to check gene expression or silencing. Forty-eight hours after transfection, the medium was collected into a 5-ml Eppendorf tube, and 250 μl of 10× trypsin was added. The cells were collected by centrifugation at 800 rpm for 5 min at 4°C and washed with 1 ml of filtrated 3 mM EDTA PBS. The pellet was resuspended in 300 μl of binding buffer [BB; 10× BB: 0.1 M Hepes (pH 7.4), 1.4 M NaCl, and 25 mM CaCl_2_] and placed in cytometry tubes. Then, 1 μl of FITC annexin V (BD Pharmagen, no. 556419) and 10 μl of FBS were added. The mix was incubated for 30 min in the dark at 4°C. After incubation, cells were washed with 1 ml of 3 mM EDTA-PBS, collected by centrifugation, and resuspended in 250 μl of 3 mM EDTA-PBS for flow cytometry. Apoptosis rate was determined in MACSQuant VYB (Miltenyi Biotec), and the results were analyzed with Flow Logic software (Miltenyi Biotec).

To analyze the apoptotic effect of KSR1 overexpression, SKMEL2 cells were plated in a T6 plate (1 million cells per well) and transfected with increasing amounts (0.5, 1, and 2 μg) of the corresponding DNAs. Forty-eight hours after transfection, the apoptosis was assessed by annexin V^+^ as previously described. In the case of APS 2-79 apoptotic effect analysis, tumor cells were plated in a T6 plate (1 million cells per well) and treated with APS 2-79 (5 μM, 48 hours), PLX4032 (10 μM 48 hours), or Staurosporine (0.5 μM 48 hours) as a positive control of apoptosis.

### Proliferation assays

Proliferation assays were performed using the PrestoBlue Cell Viability Reagent (Thermo Fisher Scientific, no. A13261). Changes in metabolic activity and, indirectly, cell number can be detected by a media color change that can be measured using absorbance-based plate readers, using 600 nm as a reference wavelength and monitoring reagent absorbance at 570 nm. To determine the effect of the silencing of IQGAP1 and KSR1 in B-RAF and N-Ras melanoma cell lines, 24 hours after transfection with the different shRNAs, the cells were counted by a Neubauer chamber or Nucleocounter (method based on propidium iodide staining). A total of 6000 cells were plated per well in three 96-well plates, one for each time point (24, 48, and 72 hours) and three replicates per condition. At the estimated time, 10 μl of room temperature PrestoBlue Reagent was added and incubated in the dark at 37°C and the absorbance was read every 30 min from 1 hour after the reagent was added.

### IC_50_ assays

APS-2-79 IC_50_ was determined using PrestoBlue reagent. Briefly, 2000 to 4000 cells were seeded in 96-well plates and treated with different drug concentrations ranging from 1 to 100 μM. Forty-eight hours after drug treatment, 10 μl of PrestoBlue Reagent was added, incubated at 37°C for 4 hours, and the colorimetric change was measured at 570 and 600 nm using a microplate reader (Thermo Fisher Scientific Multiskan FC). To correct background absorbance, control wells containing only cell culture medium (no cells) were included. IC_50_ was estimated by nonlinear regression using GraphPad7 Prism Software (www.graphpad.com/support/faq/how-to-determine-an-icsub50sub/).

### Cellular viability assays

To determine APS-2-79 effects on viability, cells were seeded in 12-well plates (25,000 cells per well) and treated with APS-2-79 (5 μM), refreshing media, and drug every day. Cell viability was assessed after 96 hours when cells were fixed with 4% formaldehyde and stained with 0.25% crystal violet. After air-drying, crystal violet stain was dissolved in 10% acetic acid. Samples from each well were transferred to a 96-well plate, and the absorbance was measured at 595 nm on a microplate reader (Thermo Fisher Scientific Multiskan FC) as an indirect measure of cell number. Results were normalized to the value of initially plated cells and plotted as a percentage of nonviable cells in each sample relative to control.

### Cellular senescence assays

Expression of pH-dependent senescence-associated β-galactosidase (SA-β-gal) activity was analyzed using the SA-β-gal staining kit (Cell Signaling Technology, no. 9860S). Senescence was induced by HRAS V12 oncogene in KSR1^−/−^ MEFs cotransfected with the KSR1 mutants in the presence or absence of shRNA for IQGAP1. Quantification of the SA-β-gal–positive cells was done using light microscopy (×63 magnification). At least 200 cells were counted per condition.

### Adipocytic differentiation assays

MEFs were seeded at 1 × 10^5^ cells per well in six-well tissue culture plates (Sarstedt). At confluency, cells were induced into adipogenesis by incubating with differentiation media (DM) containing DMEM and 10% FBS supplemented with 0.5 mM 3-isobutyl-1-methylxanthine (Sigma-Aldrich), insulin (10 μg/ml; Sigma-Aldrich), 1 μM dexamethasone (Sigma-Aldrich), and 10 μM rosiglitazone (Sigma-Aldrich). Cells were treated for 3 days (D3), followed by replacement of DM with DMEM, and 10% FBS, and incubation for another 6 days (D9). MEFs were washed with 1 ml of 1× PBS (Sigma-Aldrich) once before fixing for 5 min with 4% paraformaldehyde in PBS at room temperature. After fixation, MEFs were washed three times with PBS and once with 60% isopropanol and were completely air-dried. Triglyceride accumulation was unveiled by staining with Oil Red O (ORO; Sigma-Aldrich) for 10 min at room temperature. Excess stain residue was removed with four ddH_2_O washes. Approximately 1 ml of PBS was then added for microscopic visualization. Images were processed using a 4× objective lens, with transmitted bright-field light. For quantification, ORO stain particles were eluted with 100% isopropanol and analyzed using Thermo Fisher Scientific Varioskan Flash for spectrophotometry readings at 514 nm. Images were taken using light microscopy (×4 magnification, Leica).

### Protein purification and pull-down assays

Bacteria harboring: pGEX IQGAP1 (Fig. 2D) and pGEX KSR1 ERK BD: 301-600 (Fig. 3C) were inoculated in 50 ml of LB medium with the corresponding antibiotic resistance overnight at 37°C. The day after, this inoculum was diluted in 400 ml of LB medium and grown for 4 hours at 37°C. The recombinant protein expression was induced by adding 0.2 mM isopropyl-β-d-thiogalactopyranoside (Sigma-Aldrich no. 367-93-1) with shaking at 37°C for 3 hours. Bacteria were collected by centrifugation at 6000 rpm/10 min and the pellet was resuspended in 10 ml of 1× PBS, 1% NP-40, aprotinine (10 μg/ml), and leupeptin (10 μg/ml). They were sonicated on ice at 80% amplitude, 0.9 cycles for 7 min. The extract was centrifuged at 3500 rpm for 30 min at 4°C, and 500 μl of Glutathione-Sepharose 4B beads (GE no. 17-0618-01) was added to the supernatant. The mix was incubated with rocking at 4°C for 3 hours and washed three times with cold washing buffer, twice with cold 1× PBS, and once with MLB buffer: 25 mM Hepes (pH 7.5), 150 mM NaCl, 1% NP-40, 10% glycerol, 25 mM NaF, 10 mM MgCl_2_, 1 mM EDTA, and 1 mM sodium orthovanadate. Quantification of the proteins obtained was estimated using a BSA standard curve. Beads coated with purified GST-KSR1 301-600 were incubated with total lysates of HEK293T cells lysed in MLB buffer; beads coated with GST-IQGAP1 were incubated with His-KSR1—purified by similar procedures from bacteria expressing pHIS parallel KSR1—for 2 hours with rocking at 4°C. After incubation, the beads were washed twice with cold 1× PBS, twice with cold 1× PBS and 1% NP-40, and, lastly, twice with MLB buffer. The beads were resuspended in loading buffer 2× Laemmli and loaded in a 12% SDS-PAGE for protein analysis.

### Liquid chromatography–tandem mass spectrometry analysis

HEK293T cells were transfected with the different plasmids as indicated. The different treatments were done 24 hours after transfection. The cells were lysed in lysis buffer [1% Triton X-100, 150 mM NaCl, 20 mM tris-HCl (pH 7.5), and 1 mM EDTA (pH 7.5)]. Immunoprecipitation, washing, and digestion were performed on a KingFisher Duo robotic station (Thermo Fisher Scientific). Five microliters of magnetic antibody bead slurry, anti-HA beads (MBL bio), was diluted in 100 μl of lysis buffer and loaded in row H of a 96 deep-well plate. Five hundred microliters of lysate was loaded into row G, and 300 μl of lysis buffer was loaded into rows E and F. Three hundred microliters of wash buffer [150 mM NaCl, 20 mM tris-HCl (pH 7.5), and 1 mM EDTA (pH 7.5)] was loaded into rows B to D. Row A contained the 100 μl of digest buffer [2 M Urea, 50 mM tris-HCl (pH 7.5), 1 mM DTT, porcine trypsin (5 μg/ml; Promega), and GluC (5 μg/ml; Promega)]. The robot picked up beads in row H, transported them to row G, and released and mixed them for 2 hours. Beads were picked up and released subsequently into rows F to B with 1 min mixing in between. The washed beads were then transported into row A and digested at 27°C for 30 min under mixing. Beads were then removed and digestion continued for 8 hours at 37°C. After iodoacetamide modification and acidification of the samples, the peptide mixtures were desalted using homemade C18 tips. The desalted and lyophilized peptides were resuspended in 0.1% TFA and subjected to mass spectrometric analysis by reversed-phase nano–liquid chromatography–tandem mass spectrometry (LC-MS/MS). Mass spectrometry: 5 μl of the resuspended peptides was analyzed by reversed-phase nano–LC-MS/MS using a nano-Ultimate 3000 LC system and a QExactive plus or Lumos Fusion mass spectrometer (both Thermo Fisher Scientific). Flow rates were 400 nl/min. Peptides were loaded onto a self-packed analytical column (uChrom 1.6, 0.075 mm by 25 cm) using a 67-min gradient buffer A (2% acetonitrile, 0.5% acetic acid) and buffer B (80% acetonitrile, 0.5% acetic acid); 0 to 16 min: 2% buffer B, 16 to 56 min: 3 to 35% buffer B, 56 to 62 min: 99% buffer B; 62 to 67 min 2% buffer B. The QExactive was operated in top 12, data-dependent mode with a 30-s dynamic exclusion range. Full-scan spectra recording in the Orbitrap was in the range of *m*/*z* (mass/charge ratio) 350 to *m*/*z* 1,650 (resolution: 70,000; AGC: 3e6 ions). MS2 was performed with an isolation window of 1.4, an AGC of 5e4, an HCD collision energy of 26, and a scan range from 140- to 200-ms maximum injection time. The Lumos was operated in data-dependent mode with a 10-s dynamic exclusion range. Full-scan spectra recording in the Orbitrap was in the range of *m*/*z* 350 to *m*/*z* 1,400 (resolution: 240,000; AGC: 7.5e5 ions). MS2 was performed in the ion trap, with an isolation window of 0.7, an AGC of 2e4, an HCD collision energy of 28, rapid scan rate, a scan range of 145 to 1450 *m*/*z*, 50-ms maximum injection time, and an overall cycle time of 1 s.

Database search: The mass spectrometry raw data were analyzed by the MaxQuant and Andromeda software package (*37*) using the preselected conditions for analysis (specific proteases, two missed cleavages, and seven amino acids minimum length). Protease was set to trypsin. Carbamylation (C) was selected as fixed modification. Variable modifications were N-terminal acetylation (protein) and oxidation (M). False discovery rate was set to 0.01. MS/MS spectra were searched against the human UniProt database and the MaxQuant contaminant database with a mass accuracy of 4.5 parts per million (ppm; for MS) and 20 ppm or 0.5 Da (MS/MS OT or IT). Peak matching was selected and was limited to within 0.7 min. Elution window had a mass accuracy of 4.5 ppm.

### Statistical analyses

Statistical analyses were performed using GraphPad Prism software. Each experiment was independently repeated at least three times. All values and error bars were represented as the mean of the number of determinations with error bars representing ± SD. Two-tailed unpaired *t* tests were used to determine statistical significance between two experimental groups as indicated in the respective figure legends where the number of independent experiments (*N*) is indicated.

### Deposited data

Databases utilized: TCGA-COAD, Genomic Data Commons (https://portal.gdc.cancer.gov/projects/TCGA-COAD); TCGA-LUAD, Genomic Data Commons (https://portal.gdc.cancer.gov/projects/TCGA-LUAD); TCGA-PAAD, Genomic Data Commons (https://portal.gdc.cancer.gov/projects/TCGA-PAAD); TCGA-READ, Genomic Data Commons (https://portal.gdc.cancer.gov/projects/TCGA-READ); GSE75037, GEO (www.ncbi.nlm.nih.gov/geo/query/acc.cgi?acc=GSE75037); GSE7670, GEO (www.ncbi.nlm.nih.gov/geo/query/acc.cgi?acc=GSE7670); GSE40791, GEO (www.ncbi.nlm.nih.gov/geo/query/acc.cgi?acc=GSE40791); GSE32863, GEO (www.ncbi.nlm.nih.gov/geo/query/acc.cgi?acc=GSE32863); GSE43458, GEO (www.ncbi.nlm.nih.gov/geo/query/acc.cgi?acc=GSE43458); GSE10072, GEO (www.ncbi.nlm.nih.gov/geo/query/acc.cgi?acc=GSE10072); GSE9348, GEO (www.ncbi.nlm.nih.gov/geo/query/acc.cgi?acc=GSE9348); GSE39582, GEO (www.ncbi.nlm.nih.gov/geo/query/acc.cgi?acc=GSE39582); GSE32323, GEO (www.ncbi.nlm.nih.gov/geo/query/acc.cgi?acc=GSE32323); GSE16515, GEO (www.ncbi.nlm.nih.gov/geo/query/acc.cgi?acc=GSE16515); GSE15471, GEO (www.ncbi.nlm.nih.gov/geo/query/acc.cgi?acc=GSE15471); and GSE28735, GEO (www.ncbi.nlm.nih.gov/geo/query/acc.cgi?acc=GSE28735).

### Software and algorithms

TCGAbiolinks (*41*) (https://github.com/BioinformaticsFMRP/TCGAbiolinks); GEOquery (*42*) (www.bioconductor.org/packages/release/bioc/html/GEOquery.html); Limma (*43*) (www.bioconductor.org/packages/release/bioc/html/limma.html); and edgeR (*44*) (https://bioconductor.org/packages/release/bioc/html/edgeR.html).

## Notes

View/request a protocol for this paper from *Bio-protocol*.
