# Supplementary material for: Scaffold coupling: ERK activation by trans-phosphorylation across different scaffold protein species
Source: Sci Adv. 2023 Feb 15;9(7):eadd7969. doi: 10.1126/sciadv.add7969 (PMC9931222; doi:10.1126/sciadv.add7969)
Supplement: Supplementary file 1 — Figs. S1 to S5 [file sciadv.add7969_sm.pdf]

Science Advances
AAAS

Supplementary Materials for

Scaffold coupling: ERK activation by trans-phosphorylation across different scaffold protein species

Ana Martín-Vega *et al.*

Corresponding author: Piero Crespo, crespop@unican.es; Berta Casar, berta.casar@unican.es

Sci. Adv. **9**, eadd7969 (2023)
DOI: 10.1126/sciadv.add7969

The PDF file includes:

Figs. S1 to S5
Legend for table S1

Other Supplementary Material for this manuscript includes the following:

Table S1

SUPPLEMENTAL MATERIAL

Figure S1: IQGAP1 interaction with KSR1 (related to Fig.1)

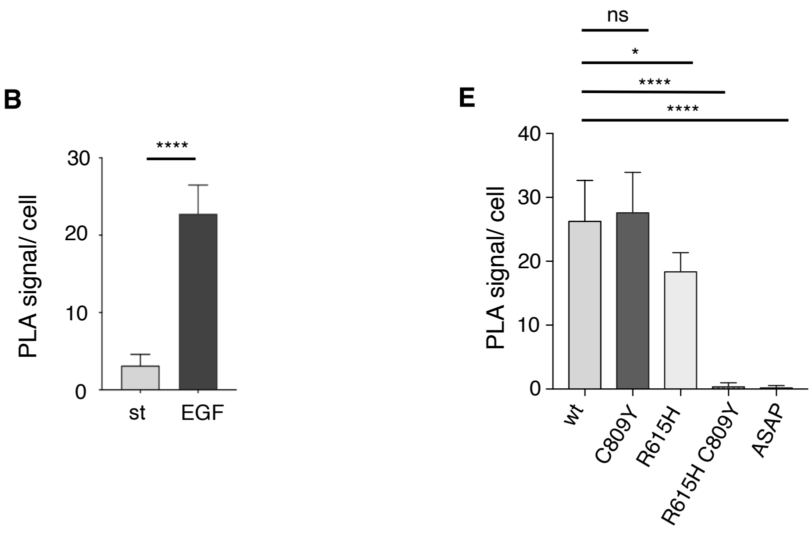

Figure S1. IQGAP1 interaction with KSR1. Quantification of the PLA dots from the data shown in Figure 1 B and E. Data shows mean ± SEM of 80-100 cells from four different fields, from 3 independent experiments. P values: * $p<0.05$; **** $p<0.001$ by two-tailed unpaired Student T-Test.

Figure S2: IQGAP1 interaction with KSR1 (related to Fig.2)

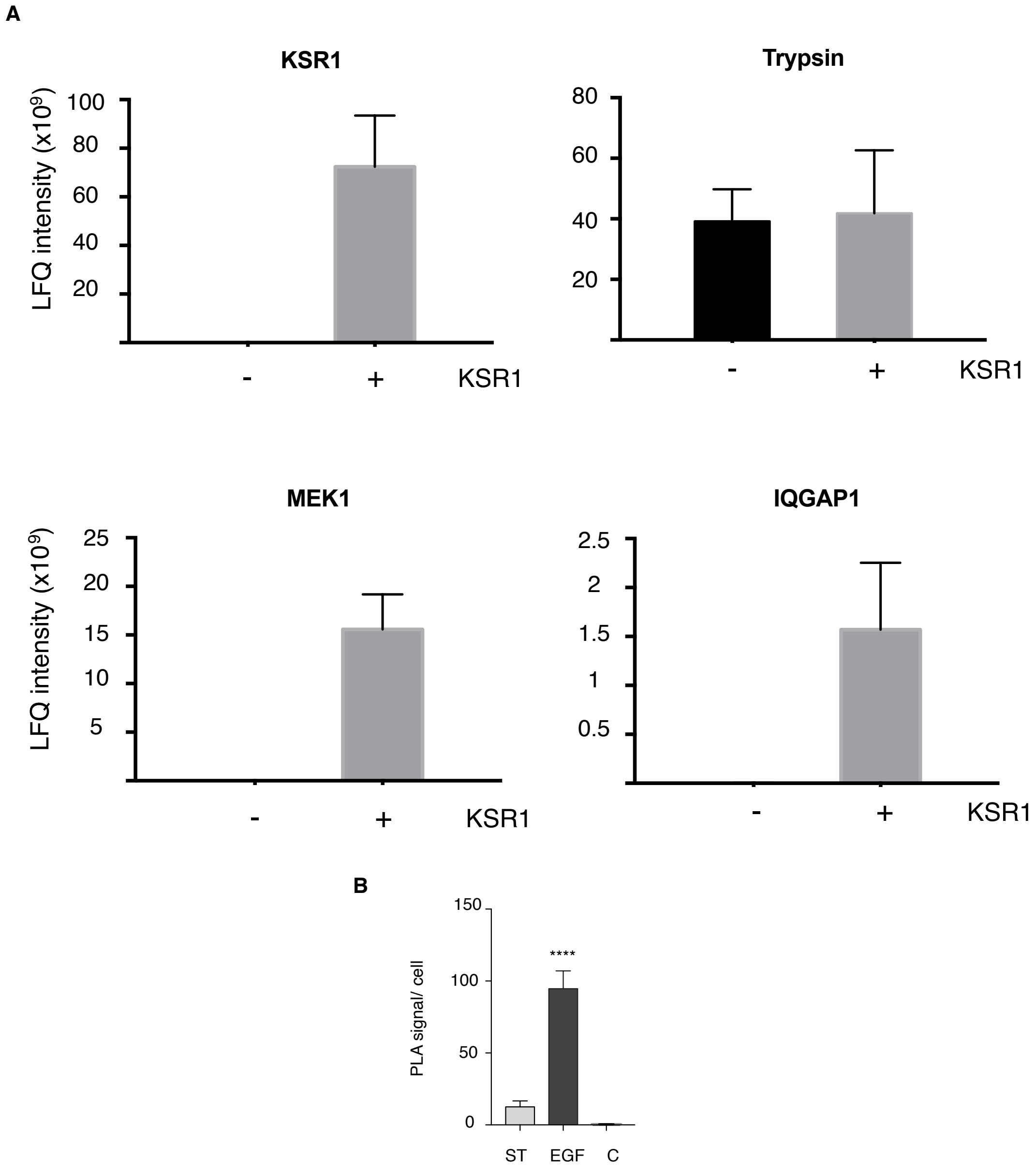

Figure S2. IQGAP1 interaction with KSR1. A) Mass spectrometry data showing interaction of different proteins in anti HA immunoprecipitates from HEK293T cells transfected with empty vector (-) or HA-KSR1 (+) and stimulated with EGF (50 ng/ml, 5min). Data represent average ± SEM LFQ intensity from three independent experiments. B) Quantification of the PLA dots from the data shown in Figure 2E. Data shows mean ± SEM of 80-100 cells from four different fields, from 3 independent experiments. P values: *** p<0.001 by two-tailed unpaired Student T-Test.

Figure S3: KSR1 and IQGAP1 binding determinants (related to Fig.3)

A

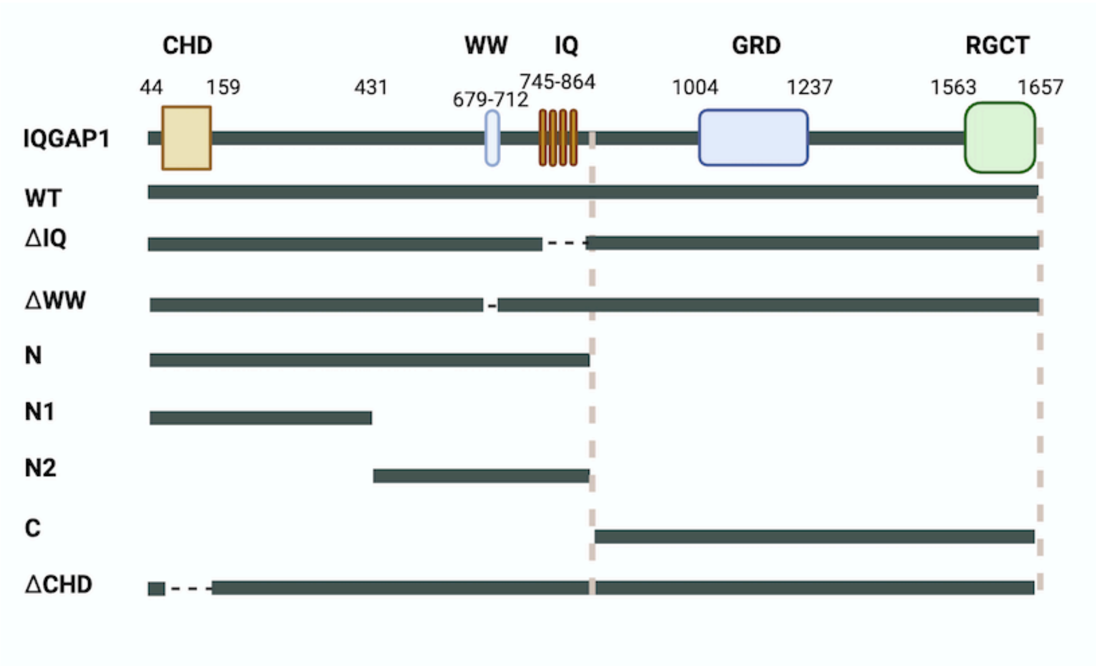

B

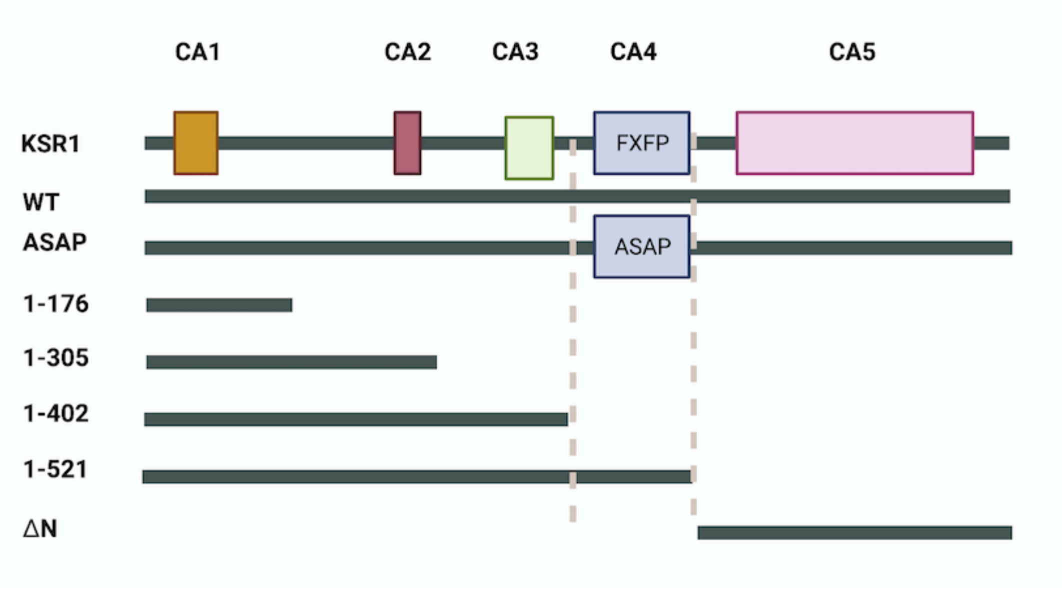

Figure S3. KSR1 and IQGAP1 binding determinants. Depiction of the IQGAP1 (A) and KSR1 (B) mutants utilized in the experiments described in Figure 3. The regions enclosed by the red hatched lines indicate their respective binding regions.

Figure S4: The negative correlation in transcript levels among *KSR* and *IQGAP* is restricted to specific family members (Related to Fig. 7)

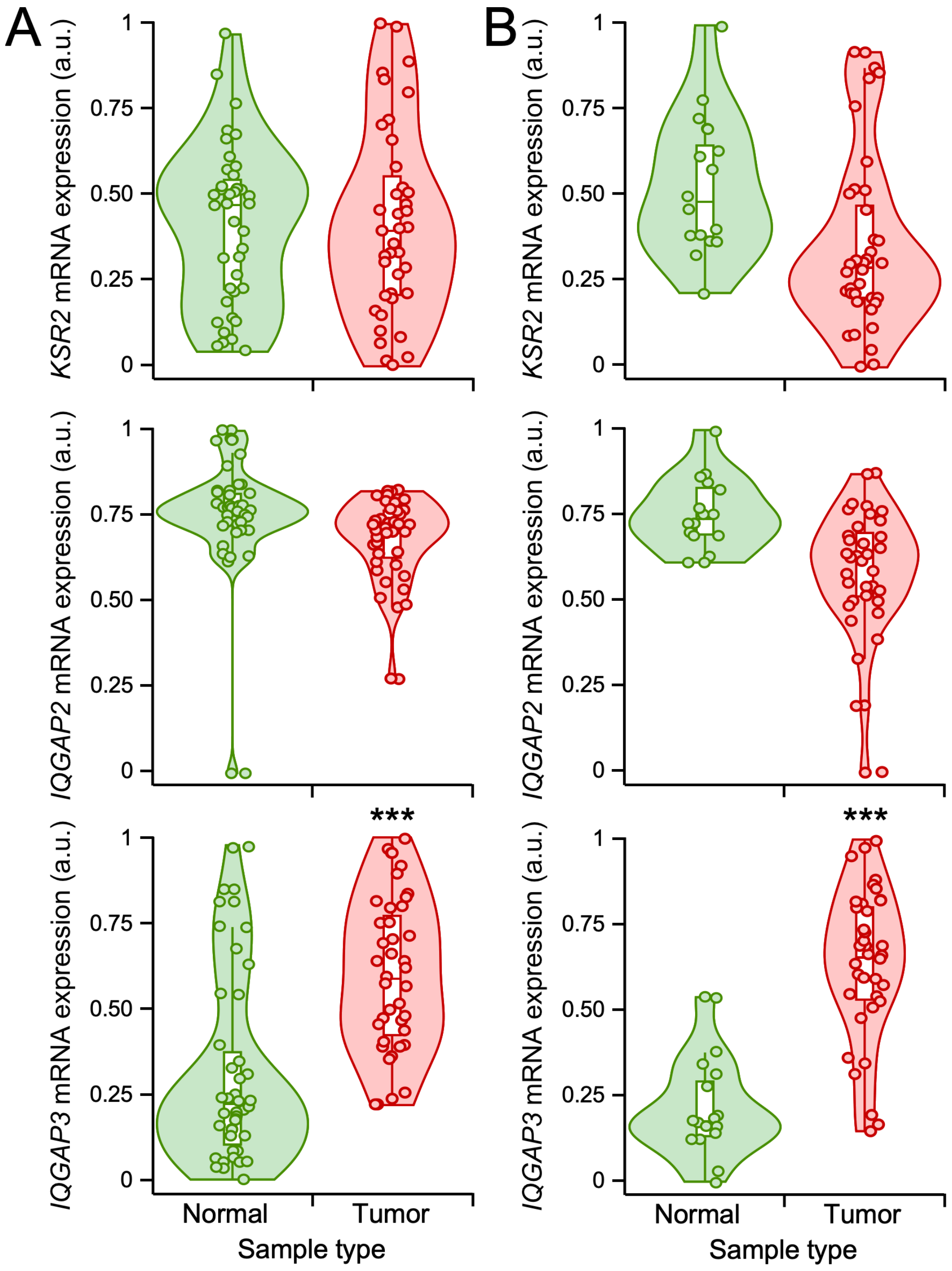

Figure S4. The negative correlation in transcript levels among *KSR* and *IQGAP* is restricted to specific family members. A, B) Relative expression levels of *KSR2* (A, B; top panels), *IQGAP2* (A, B; middle panels) and *IQGAP3* (A,B; bottom panels) transcripts in healthy and tumoral samples obtained from the microarray datasets GSE15471 (A) and GSE16515 (B). Data representation has been done as in Figure 6. ***, $P < 0.001$.

Figure S5: Effects of APS-2-79 on cell viability in NRAS-mutant tumour cell lines (related to Fig. 8).

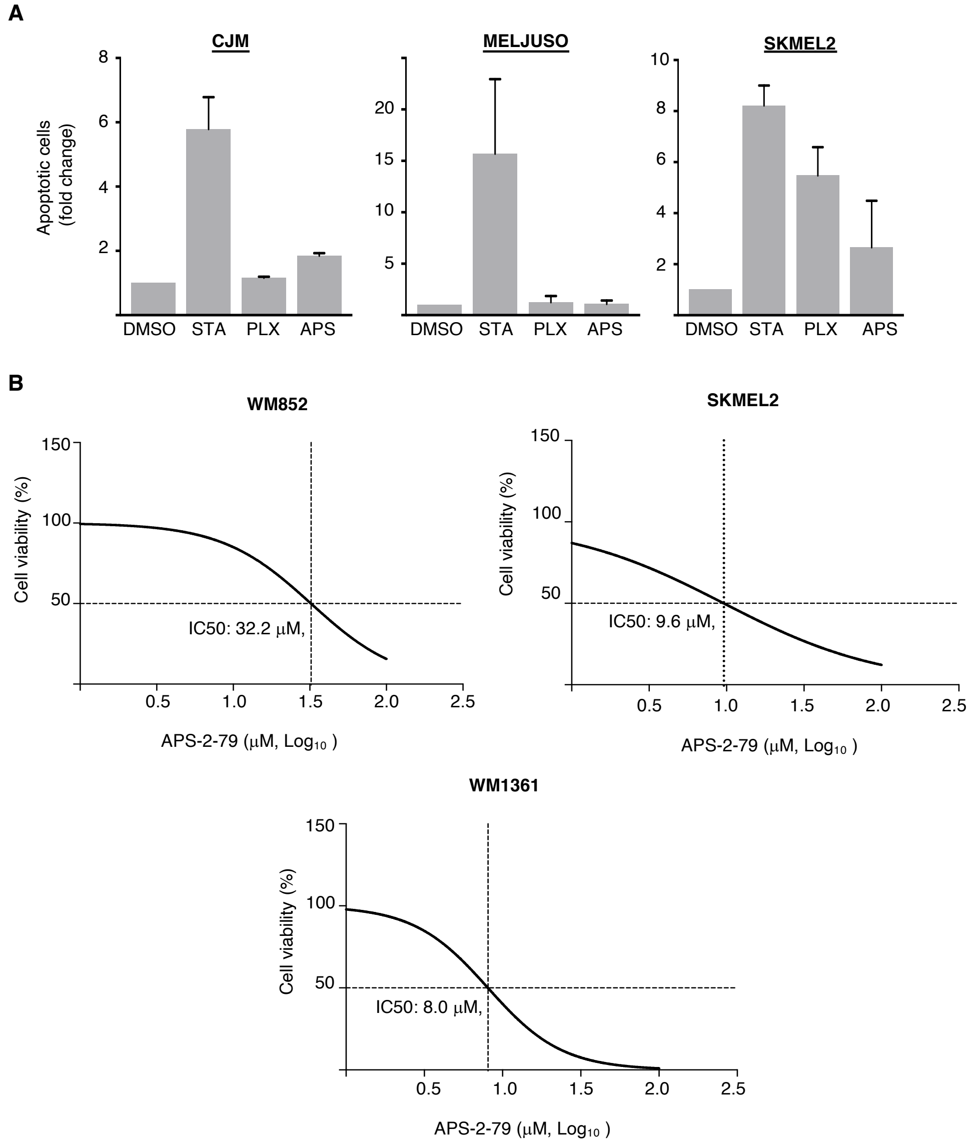

Figure S5 Effects of APS-2-79 on cell viability in NRAS-mutant cell lines. A) The indicated cell lines were treated for 48 h with APS-2-79 (5 µM), PLX4032 (10 µM) or staurosporine (0.5 µM). Apoptosis was evaluated by annexin V levels using MACSQuant cytometer and results were analysed with FlowLogic software. Results show mean ± SEM of three independent experiments normalized to the levels of control (DMSO treated) cells. **B)** IC_{50} values for the indicated cell lines. Data shows average of three independent experiments.

Supplementary Excel Table S1. Expression of *KSR1* and *IQGAP1* mRNAs in different datasets.
